# Effects of Maraviroc and Efavirenz on Markers of Immune Activation and Inflammation and Associations with CD4^+^ Cell Rises in HIV-Infected Patients

**DOI:** 10.1371/journal.pone.0013188

**Published:** 2010-10-06

**Authors:** Nicholas Funderburg, Magdalena Kalinowska, James Eason, James Goodrich, Jayvant Heera, Howard Mayer, Natasa Rajicic, Hernan Valdez, Michael M. Lederman

**Affiliations:** 1 Case Western Reserve University, Cleveland, Ohio, United States of America; 2 ViiV Healthcare, Research Triangle Park, North Carolina, United States of America; 3 Pfizer Global Research and Development, New London, Connecticut, United States of America; 4 EMD Serono, Rockland, Massachusetts, United States of America; 5 Pfizer Inc, New York, New York, United States of America; University of Cape Town, South Africa

## Abstract

**Background:**

Maraviroc treatment for HIV-1 infected patients results in larger CD4^+^ T cell rises than are attributable to its antiviral activity alone. We investigated whether this is due to modulation of T cell activation and inflammation.

**Methods and Findings:**

Thirty maraviroc-treated patients from the Maraviroc versus Efavirenz Regimens as Initial Therapy (MERIT) study were randomly selected from among those who had CCR5-tropic (R5) HIV on screening and achieved undetectable HIV RNA (<50 copies/mL) by Week 48. Efavirenz-treated controls were matched for baseline characteristics to the maraviroc-treated patients selected for this substudy. Changes in immune activation and inflammation markers were examined for associations with CD4^+^ T cell changes. Maraviroc treatment tended to result in more rapid decreases in CD38 expression on CD4^+^ T cells and in plasma D-dimer concentrations than did treatment with efavirenz. The proportion of patients with high-sensitivity C-reactive protein >2 µg/mL increased from 45% to 66% in the efavirenz arm, but remained constant in the maraviroc arm (*P* = 0.033). Decreases in CD38 expression on CD8^+^ T cells were correlated with CD4^+^ T cell rises for maraviroc treatment (r = −0.4, *P* = 0.048), but not for treatment with efavirenz.

**Conclusions:**

Maraviroc-treated patients had earlier, modest decreases in certain markers of immune activation and inflammation, although in this small study, many of the differences were not statistically significant. Levels of high-sensitivity C-reactive protein remained constant in the maraviroc arm and increased in the efavirenz arm. Decreases in immune activation correlated with increased CD4^+^ T cell gains.

**Trial Registration:**

ClinicalTrials.gov NCT00098293

## Introduction

Chronic HIV infection is characterized by persistent immune activation, as measured by CD38 expression on T cells [1]–[3]. This immune activation has been associated with CD4^+^ T cell loss and disease progression [1]–[4]. After adequate control of HIV replication, the level of immune activation does not return to the levels observed in uninfected adults [5]. This residual heightened T cell surface expression of CD38 has been associated with lower CD4^+^ T cell gains following highly active antiretroviral therapy (HAART) [5].

Increased plasma levels of inflammatory markers have also been found in patients with appropriately treated HIV disease [6]. Inflammation has been linked to morbidity and mortality in HIV infection. In the Strategies for Management of Antiretroviral Therapy (SMART) study, increased levels of the pro-inflammatory cytokine interleukin 6 (IL-6) and of D-dimer, a marker of fibrinolysis, predicted all-cause mortality in persons with treated HIV infection, and impaired liver function among hepatitis co-infected patients [7], [8]. Levels of IL-6 and high-sensitivity C-reactive protein (hsCRP) were also independent predictors of cardiovascular events and AIDS-related opportunistic infections [8]–[10]. In addition, studies in non-HIV-infected populations have shown that there may be a clinical benefit associated with reducing inflammation in persons with elevated levels of these markers: In the Justification for the Use of Statins in Prevention: an Intervention Trial Evaluating Rosuvastatin (JUPITER), individuals with hsCRP >2.0 µg/mL and normal blood lipids who were treated with rosuvastatin had a significantly reduced incidence of major cardiovascular events [11]. This suggests that HIV-infected patients with elevated markers of inflammation and immune activation may also benefit from reduction of inflammation.

In the phase 3 MERIT study comparing the CCR5 antagonist maraviroc (MVC) to the non-nucleoside reverse transcriptase inhibitor efavirenz (EFV), patients who received MVC had significantly greater increases in blood CD4^+^ T cells than those who received EFV, although a numerically higher proportion of EFV-treated patients achieved undetectable (<50 copies/mL) HIV RNA [12]. To determine whether the greater CD4^+^ T cell recovery on MVC might be related to a differential effect on cellular activation and inflammation, we assayed stored samples from a subset of patients from each arm of MERIT for markers of cellular activation and plasma inflammatory markers. When analyzing the inflammatory marker hsCRP, we selected 2.0 µg/mL as a threshold level based on results of the JUPITER study that suggested a clinical benefit to reducing inflammation for individuals with hsCRP >2.0 µg/mL.

## Materials and Methods

**Figure 1:**
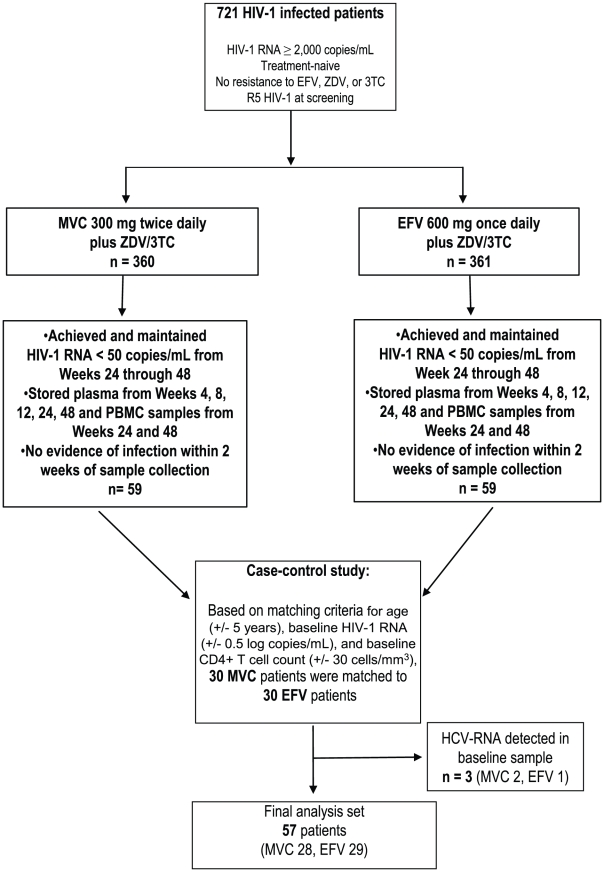
Patient flow chart: MERIT Immune Activation Sub-analysis. Notes under Figure 1: *MVC, maraviroc; EFV, efavirenz; ZDV, zidovudine; 3TC, lamivudine.*

As described elsewhere [12], MERIT evaluated the virologic and immunologic effects of MVC versus EFV, both given with zidovudine/lamivudine, in treatment-naive patients with CCR5-tropic (R5) HIV infection. MERIT participants were asked to consent to storing blood samples for future research, and only samples from patients who consented were analyzed. The MERIT study, including written informed consent for stored specimens, was approved by the Institutional Review Boards (IRB) or Independent Ethics Committees (IEC) of all participating sites. The protocol for the MERIT study is available as Protocol S1. Table S1 lists the 24 IRBs and IECs responsible for patients in the analysis described here. The current sub-analysis enrolled 30 MVC-treated patients randomly selected from among those who achieved and maintained HIV RNA <50 copies/mL between weeks 24 and 48, had available plasma at all time points when markers were assayed (weeks 4, 8, 12, 24, and 48) and peripheral blood mononuclear cell (PBMC) samples at weeks 24 and 48, had no documented or reported infections for 2 weeks prior to each time point, and no evidence of HCV infection (Figure 1). EFV-treated controls were matched to cases for age, baseline CD4^+^ cell count, and baseline HIV RNA. Each patient sample was assigned a numerical code, and the laboratory personnel performing all assays were blinded to the treatment arm from which each sample was collected.

Frozen PBMCs were thawed in batch and T cells were identified by size, granularity, and by positive staining with Peridinin-chlorophyll-protein Complex (PerCP)-labeled murine monoclonal antibody to CD3 and either allophycocyanin (APC)-labeled antibody to CD4, or fluorescein isothiocyanate (FITC)-labeled antibody to CD8. Electronically gated CD4^+^ or CD8^+^ T lymphocytes were then analyzed for expression of CD38 with a phycoerythrin (PE)-labeled antibody. CD38 expression is reported as antibodies bound per cell. Appropriately conjugated murine monoclonal isotype control antibodies were used for gating. All antibodies were purchased from BD Bioscience, San Diego, CA. Cells were stained for 10 min at room temperature, washed in phosphate buffered saline with 1% bovine serum albumin and 0.1% sodium azide, then fixed in 1% formaldehyde and analyzed using a dual-laser flow cytometer (FACSCaliber; Becton Dickinson, San Jose, CA) and CellQuest software (BD Bioscience). Plasma was prepared by centrifugation of whole blood collected into EDTA-containing tubes at 1500–2000×g for 10–15 minutes and was stored at −80°C until analysis. Plasma samples were thawed and analyzed in batch. Levels of D-dimers (Asserachrom D-DI immunoassay, Diagnostica Stago, Asnières sur Seine, France), tumor necrosis factor receptor I (TNFRI) (R&D Systems, Minneapolis, MN), TNFRII (Biosource, Nivelles, Belgium), Neopterin (Alpco Diagnostics, Salem, NH), IL-6 (R&D Systems), and hsCRP (United Biotec Incorporated, Mountain View, CA), were measured by ELISA.

Immune activation markers were assayed over time and changes reported as median and interquartile range. Differences in percent change from baseline to week 24 and 48 between the two arms were analyzed using a Wilcoxon Rank Sum test. A Cochran-Mantel-Haenszel test was used to compare the treatment arms with respect to a number exceeding threshold at week 48 while adjusting for baseline status. Changes from baseline to week 48 of each marker were analyzed for association with change from baseline to week 48 in CD4^+^ cell count using Pearson's correlation. The statistical analysis plan was developed and specified prior to any execution of the study. We did not consider adjustment for multiple comparisons in this exploratory, hypothesis-generating set of analyses.

## Results

Thirty randomly selected patients who had received MVC were matched to 30 patients who had been treated with EFV based on age, baseline CD4^+^ T cell count, and baseline HIV RNA levels. All patients selected for this sub-study had reached undetectable (<50 copies/mL) viral levels in plasma between weeks 24 and 48. Complete data sets were available for 28 MVC-treated and 29 EFV-treated patients. Most patients were male (EFV 72%, MVC 82%) and the median age of each group was 36. At baseline, HIV RNA levels were 4.9 log_10_ copies/mL and CD4^+^ lymphocyte counts were 274 cells/µL in both treatment groups. Baseline levels of immune activation and inflammation markers were similar across the two groups (Table 1).

**Table 1:** Markers of immune activation and inflammation at baseline.

Immunologic marker	EFV + ZDV/3TC (n = 29)	MVC + ZDV/3TC (n = 28)
CD4^+^ activation (CD38 antibodies/cell), median (IQR)	1397 (927, 1809)	1395 (1049, 2071)
Percentage of activated CD4^+^ cells (% CD38^+^), median (IQR)	76.8 (64.9, 84.9)	74.3 (65.7, 82.2)
CD8^+^ activation (CD38 antibodies/cell), median (IQR)	1925 (1117, 3001)	1878 (1533, 3165)
Percentage of activated CD8^+^ cells (% CD38^+^), median (IQR)	81.4 (67.8, 89.4)	81.9 (78.3, 88.9)
D-dimer, ng/mL, median (IQR)	89.4 (61.1, 208.7)	115.5 (71.2, 275.7)
IL-6, pg/mL, median (IQR)	1.9 (0.8, 2.9)	1.7 (1.1, 2.5)
hsCRP, µg/mL, median (IQR)	1.6 (1.0, 4.2)	1.4 (0.9, 3.4)
Proportion with hsCRP >2 µg/mL	44.8	35.7

IQR, interquartile range (25^th^–75^th^ percentile); MVC, maraviroc; EFV, efavirenz; ZDV/3TC, zidovudine/lamivudine.

**Figure 2:**
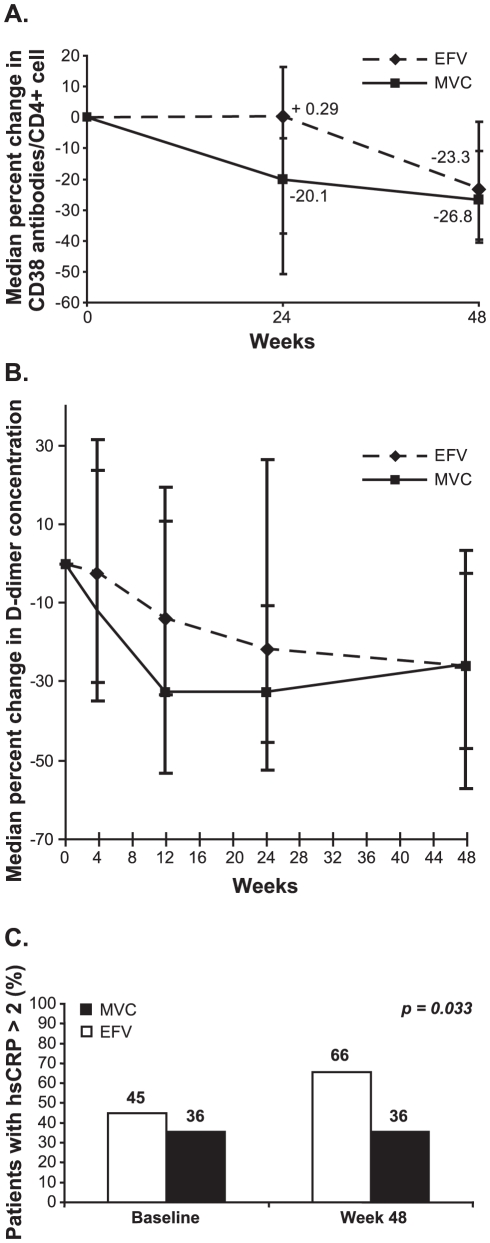
Treatment effects on markers of immune activation and inflammation. Among maraviroc (MVC)-treated patients, there was an earlier decrease in CD38 expression on CD4^+^ T cells (A), and plasma levels of D-dimers tended to decrease more rapidly (B). The proportion of efavirenz (EFV)-treated patients with hsCRP >2 µg/mL increased while the proportion of MVC-treated patients with hsCRP>2 µg/mL remained constant (C). Vertical lines represent interquartile ranges (IQR; 25^th^–75^th^ percentile).

At week 48, MVC-treated patients had a mean change in CD4^+^ T cell count of +212 cells/µL compared to +191 cells/µL for the EFV group. In order to determine whether the increased restoration of CD4^+^ T lymphocytes in the MVC treated group was related to decreases in lymphocyte activation, surface expression of CD38 on T cells was measured at baseline, 24 and 48 weeks. Among patients treated with MVC, there was an earlier decrease in CD38 expression on CD4^+^ T cells than was seen among EFV-treated patients, although this difference was not statistically significant. There was a 20.1% decrease in CD38 levels (as measured by antibody binding) on CD4^+^ cells at week 24 versus a 0.29% increase in CD38 levels in the EFV arm (*P* = 0.137; Figure 2A). The decreases in CD38 levels on CD8^+^ T cells were almost identical in the two arms (data not shown).

Plasma levels of D-dimers also tended to fall more rapidly among patients treated with MVC, although the difference between the arms was not significant (*P* = 0.177 at Week 24; Figure 2B). Between baseline and week 48, the proportion of EFV-treated patients with hsCRP >2 µg/mL increased (from 45% to 66%) while the proportion of MVC-treated patients with hsCRP >2 µg/mL remained constant (36%) (Figure 2C; *P* = 0.033). Levels of plasma IL-6 fell more quickly in the EFV arm, but this difference did not approach significance (*P* = 0.817 at Week 24; *P* = 0.448 at Week 48). Declines in plasma levels of soluble TNFRI, soluble TNFR II, and neopterin were similar for the MVC-treated and EFV-treated groups (data not shown).

Among MVC-treated patients, decreases in CD38 expression on CD8^+^ T cells were significantly correlated with the rise in blood CD4^+^ T cells (r = −0.4, *P* = 0.048,Table 2). This correlation was not observed among EFV-treated patients. When both treatment arms were combined, significant correlations were observed between gains in CD4^+^ T cells and decreases in CD38 expression on both CD4^+^ and CD8^+^ T cells (Table 2).

**Table 2:** Correlations of changes in immune markers with changes in CD4^+^ cell count.

Immunologic marker	Correlation Coefficient (*P*-value)		
	EFV + ZDV/3TC (n = 29)	MVC + ZDV/3TC (n = 28)	Combined groups (n = 57)
CD4^+^ activation (CD38 antibodies/cell)	−0.3 (0.10)	−0.3 (0.11)	−0.3 (0.01)
Percentage of activated CD4^+^ cells (% CD38^+^)	−0.3 (0.17)	−0.2 (0.27)	−0.3 (0.06)
CD8^+^ activation (CD38 antibodies/cell)	−0.2 (0.23)	−0.4 (0.048)	−0.3 (0.02)
Percentage of activated CD8^+^ cells (% CD38^+^)	−0.3 (0.10)	−0.2 (0.25)	−0.3 (0.03)
D-dimer	−0.2 (0.41)	0.09 (0.64)	−0.08 (0.55)
IL-6	−0.2 (0.37)	0.06 (0.77)	−0.06 (0.68)
hsCRP	0.13 (0.51)	0.2 (0.29)	0.16 (0.22)

MVC, maraviroc; EFV, efavirenz; ZDV/3TC, zidovudine/lamivudine.

## Discussion

Treatment with MVC has resulted in larger increases in CD4^+^ T cell counts than can be attributed to its antiviral effects alone [12], [13], and the mechanism responsible has not been identified. In the current sub-analysis, MVC-treated patients demonstrated a larger mean increase in CD4^+^ T cells than EFV-treated patients, consistent with the results of the larger MERIT study [12]. The increase in CD4^+^ T cell numbers associated with CCR5 inhibition could be a simple consequence of blocking the trafficking of CCR5^+^ T cells between circulation and peripheral tissues. Alternatively, interference with signaling via CCR5 antagonism might more rapidly attenuate the immune activation that has been linked to impaired CD4^+^ T cell restoration in HIV infection [4], [5], [14]. Here, we measured levels of inflammation, coagulation, and cellular activation to determine whether the CD4^+^ T cell rises associated with MVC administration were related to modulation of immune activation. Compared to patients receiving EFV, patients receiving MVC tended to have earlier decreases in CD38 expression on their CD4^+^ T cells. Decreases in CD38 expression on CD8^+^ T cells correlated with increases in CD4^+^ T cell counts in the MVC arm, but not in the EFV arm. When both arms were combined, however, decreases in CD38 expression on both CD4^+^ and CD8^+^ T cells were linked to the magnitude of CD4^+^ T cell restoration. When taken together, this small data set, while not conclusive, provides a preliminary indication that the enhanced CD4^+^ T cell restoration seen in patients treated with MVC may be related more to interfering with immune activation pathways than with cellular redistribution.

Treatment with MVC has effects on activation and inflammation in HIV infection that can be differentiated from the effects of another antiretroviral treatment regimen. D-dimer levels tended to fall more rapidly in MVC-treated patients, while levels of IL-6 tended to drop slightly earlier in patients treated with EFV. Levels of CRP remained stable in the MVC arm and tended to increase in patients in the EFV arm. The inflammatory marker CRP, when measured with high sensitivity assays, is a known predictor of cardiovascular events in the general population [15], [16], and among treated HIV-infected patients, has been associated with cardiovascular events [10], [17] and opportunistic infections [9]. Levels of CRP were assessed using a threshold value of 2 µg/mL based on previous research indicating that individuals with a value exceeding this level may have an increased risk of cardiovascular events [18], [19] and may benefit from reduction of inflammation (11). More broadly, indices of inflammation and coagulation are linked to AIDS-defining and non-AIDS-defining events and mortality [7], [20]. In recent studies, levels of inflammatory markers in HIV-infected persons have been associated with internal carotid intima-media thickness, a surrogate marker for cardiovascular disease [21], [22], consistent with a growing appreciation in other settings that chronic inflammation and its effects on coagulation may be important drivers of cardiovascular risk [11], [15], [16], [18], [19]. Since viral replication drives immune activation, initiation of therapy in either arm caused an early decrease in viral load accompanied by a decline in inflammation. The early, modest differential effect of MVC on several markers suggests an additional direct effect of the drug on immune activation and inflammation. In addition, CCR5 has been implicated as a determinant of cardiovascular risk in murine atherosclerosis models [23] and in patients undergoing hemodialysis [24]. Whether the changes induced by MVC will be associated with fewer inflammation-associated events among patients receiving HAART, such as immune reconstitution inflammatory syndrome and cardiovascular disease, is being explored in controlled trials.

## Supporting Information

Table S1 Independent ethics committees and institutional review boards approving the MERIT study for patients in the immune activation sub-analysis.
(0.06 MB DOC)

Protocol S1 (0.57 MB DOC)
