# Supplementary material for: Effects of Maraviroc and Efavirenz on Markers of Immune Activation and Inflammation and Associations with CD4+ Cell Rises in HIV-Infected Patients
Source: PLoS One. 2010 Oct 6;5(10):e13188. doi: 10.1371/journal.pone.0013188 (PMC2950842; doi:10.1371/journal.pone.0013188)
Supplement: Table S1 — Independent ethics committees and institutional review boards approving the MERIT study for patients in the immune activation sub-analysis. (0.06 MB DOC) [file pone.0013188.s001.doc]

**Table S1**. Independent Ethics Committees and Institutional Review Boards Approving the MERIT Study for Patients in the Immune Activation Sub-Analysis

| *Institutional Review Board or*  *Independent Ethics Committee* | *Institution* | *Location* |
| --- | --- | --- |
| Ethisch Comite UZ Gent | Heymans Instituut | Gent, Belgium |
| Comite Local d'Ethique Hospitalier | CHU St Pierre/UMC St Pieter | Bruxelles, Belgium |
| Sunnybrook and Women’s College | Sunnybrook and Women’s College Health Sciences Center | Toronto, Canada |
| University of Alberta Health Research Ethics Board | University of Alberta | Edmonton, Canada |
| University of Manitoba Bannatyne Campus Research Ethics Board | University of Manitoba | Winnipeg, Canada |
| Comitato Etico Fondazione Centro San Raffaele del Monte Tabor | Istituto Scientifico Ospedale San Raffaele | Milano, Italy |
| Komisja Bioetyczna przy Centrum Medycznym Ksztalcenia Podyplomowego | Centrum Medycznym Ksztalcenia Podyplomowego | Warszawa, Poland |
| Pharma Ethics (Pty) Ltd | Lyttleton Manor | Pretoria, South Africa |
| WITS Health Consortium  Human Research Ethics Committee | WITS Health Consortium | Johannesburg, South Africa |
| University of Stellenbosch  Faculty of Health Science, Pharmaceutical Trial Advisory Committee | University of Stellenbosch  Faculty of Health Science, | Cape Town, South Africa |
| SPUK fuer Innere Medizin | UniversitaetsSpital Zuerich | Zuerich, Switzerland |
| South East Multi Centre Research Ethics Committee | Kent & Medway Health Authority | Kent, United Kingdom |
| Lothian Research Ethics Committee | Deaconess House | Edinburgh, United Kingdom |
| Brighton & Hove Research Ethics Committee | Brighton & Hove Primary Care Trust | Brighton, United Kingdom |
| King’s College Hospital Research  Ethics Committee | King's College Hospital | London, United Kingdom |
| Royal Free Hospital and Medical School Research Ethics Committee | Royal Free Hospital | London, United Kingdom |
| Kaiser Permanente Northern California Institutional Review Board | Kaiser Foundation Research Institute | Oakland, California, USA |
| Schulman Associates IRB, Inc. |  | Cincinnati, Ohio, USA |
| Western Institutional Review Board |  | Olympia, Washington, USA |
| Community Research Risk Protection Committee | Houston Clinical Research Network | Houston, Texas, USA |
| Tufts-New England Medical Center | Tufts-New England Medical Center | Boston, Massachusetts, USA |
| IUPUI Institutional Review Board | Indiana University | Indianapolis, Indiana, USA |
| Colorado Multiple Institutional Review Board |  | Aurora, Colorado, USA |
| University of Cincinnati Medical Center Institutional Review Board | University of Cincinnati Medical Center | Cincinnati, Ohio, USA |
