# Supplementary material for: Effects of Maraviroc and Efavirenz on Markers of Immune Activation and Inflammation and Associations with CD4+ Cell Rises in HIV-Infected Patients
Source: PLoS One. 2010 Oct 6;5(10):e13188. doi: 10.1371/journal.pone.0013188 (PMC2950842; doi:10.1371/journal.pone.0013188)
Supplement: Protocol S1 — (0.57 MB DOC) [file pone.0013188.s002.pdf]

CLINICAL PROTOCOL

A MULTICENTER, RANDOMIZED, DOUBLE-BLIND, COMPARATIVE TRIAL OF A NOVEL CCR5 ANTAGONIST, UK-427,857, IN COMBINATION WITH ZIDOVUDINE/LAMIVUDINE VERSUS EFAVIRENZ IN COMBINATION WITH ZIDOVUDINE/LAMIVUDINE FOR THE TREATMENT OF ANTIRETROVIRAL-NAÏVE HIV-1 INFECTED SUBJECTS

**Compound:**	UK-427,857
**Compound Name:**	
**US IND Number:**	65,229
**Protocol Number:**	A4001026
**Phase:**	Phase 2b/3
**Version and Date:**	Version 1
	24 August 2004

0100000427327\1.0\Approved\24-Aug-2004 11:39

This document contains confidential information belonging to Pfizer. Except as otherwise agreed to in writing, by accepting or reviewing this document, you agree to hold this information in confidence and not to disclose it to others (except where required by applicable law) or to use it for unauthorized purposes. In the event of any actual or suspected breach of this obligation, Pfizer should be promptly notified.

SUMMARY

INDICATION:

HIV-1 treatment in combination with other agents.

RATIONALE:

The rationale for studying UK-427,857 for the treatment of HIV-1 infection is based on the fact that persons who are heterozygous for the Δ32 mutant CCR5 allele (ie, fewer functional CCR5 receptors) have lower serum viral loads, a better response to highly active antiretroviral therapy (HAART) and delayed progression to AIDS or death.[1,2] Treatment-naïve patients are arguably the most appropriate patient population to study, since the chemokine receptor CCR5 is the primary co-receptor for nonsyncytium inducing HIV-1, which predominates during the early stages of HIV-1 infection.[3] While the number of approved antiretroviral agents has increased substantially over the past decade, there remain fundamental problems with members of all four major classes including low genetic barrier to resistance (non-nucleoside reverse transcriptase inhibitors), cross-class resistance (all), mitochondrial toxicity (nucleoside reverse transcriptase inhibitors), lipodystrophy (protease inhibitors) and mode of administration (fusion inhibitors).

Complicating matters even further, the prevalence of transmitted virus resistant to existing antiretroviral agents is increasing. Thus, there is a clear need for new drugs with novel mechanisms of action to be developed for treatment-naïve patients. UK-427,857, a selective and reversible CCR5 co-receptor antagonist, has been shown to be active in vitro against a wide range of clinical isolates (including those resistant to existing classes). In HIV-1 infected patients, UK-427,857 given as monotherapy for 10 days reduced HIV-1 viral load by up to 1.6 $\log_{10}$, consistent with currently available agents comprising the cornerstone of HAART (eg, protease inhibitors, non-nucleoside reverse transcriptase inhibitors). An excellent safety and toleration profile has been demonstrated with dosing for up to 28 days at 300 mg bid, including no significant effect on QTc interval. Since the vast majority of patients who are antiretroviral-naïve are infected with CCR5-tropic virus only, studies with UK-427,857 in this patient population are warranted.

OBJECTIVES:

- The primary objective is to confirm the hypothesis that UK-427,857 is not inferior to efavirenz, when used in combination with zidovudine/lamivudine, for the treatment of antiretroviral-naïve HIV-1 infected subjects as measured by the percentage of subjects with HIV-1 RNA below the limits of assay detection (fewer than 400 and fewer than 50 copies of HIV-1 RNA per milliliter of plasma) at 48 weeks.
- The secondary objectives include assessments of safety and tolerability of UK-427,857 versus efavirenz, when added to zidovudine/lamivudine.

090000042732721.0\Approved\24-Aug-2004 11:39

TRIAL DESIGN:

Multi-center, double blind, randomized (1:1:1), comparative non-inferiority Phase 2b/3 hybrid (run-in) study to determine the antiviral activity and safety of UK-427,857 (300 mg once daily and 300 mg twice daily) added to zidovudine/lamivudine (300 mg/150 mg twice daily), compared to efavirenz (600 mg once daily) added to zidovudine/lamivudine (300 mg/150 mg twice daily), in CCR5-tropic, HIV-1 infected subjects. This study will involve approximately 140 centers from around the world to achieve a total randomized subject population of 1071 subjects.

Patients eligible for enrollment will be males or females at least 16 years of age, with documented HIV-1 infection and more than 2000 copies of HIV-1 RNA per milliliter of plasma (measured by Roche Amplicor HIV-1 Monitor, version 1.5) and CCR5 co-receptor viral tropism as determined by the PhenoSense™ HIV entry assay (ViroLogic, Inc, South San Francisco, CA). Major exclusion criteria would include more than 14 days of treatment with any antiretroviral therapy, treatment for an opportunistic infection within 30 days prior to screening, suspected primary HIV-1 infection and/or evidence of X4- or dual-tropic virus or repeated assay failure at baseline. All subjects will undergo genotypic and phenotypic testing for the presence of non CCR5-tropic HIV-1 and for resistance to reverse transcriptase inhibitors to determine eligibility. An interim analysis of time averaged difference in viral load and response rate (percentage with HIV-1 RNA less than 400 copies/mL) on 204 patients after 16 weeks will be performed and the results reviewed by the Data Safety Monitoring Board (DSMB). The Investigators, patients and Pfizer personnel directly involved in the conduct of the study will remain blinded to this analysis. The percentage of patients with HIV-1 RNA less than 400 copies/mL will be assessed at 24, 48 and 96 weeks or at the time of discontinuation from the study. All subjects who receive at least one dose of study medication will be assessed for safety. The study will enroll over approximately an 18-month period (5 months run-in, 13 months Phase 3) with 96 weeks of treatment. The primary efficacy endpoint will be assessed after 48 weeks of treatment. As part of this clinical study a blood sample will be taken for non-anonymized pharmacogenetic analysis.

0100000427327 2\1.0\Approved\24-Aug-2004 11:39

ENDPOINTS:

Primary Endpoint:

The percentage of patients with fewer than 400 copies and the percentage of patients with fewer than 50 copies of HIV-1 RNA per milliliter of plasma at 48 weeks.

Secondary Endpoints:

The change from baseline in $\log_{10}$ transformed HIV-1 RNA levels, Time-Averaged Difference (TAD) in $\log_{10}$ transformed HIV-1 RNA levels, time to loss of virological response, change in CD4 and CD8 counts, association between baseline resistance and virological response and changes in genotype, phenotype and/or tropism in treatment failures after Week 4.

TRIAL TREATMENTS:

1. Zidovudine 300 mg PO twice daily + Lamivudine 150 mg PO twice daily + UK-427,857 300 mg PO taken once daily.
2. Zidovudine 300 mg PO twice daily + Lamivudine 150 mg PO twice daily + UK-427,857 300 mg PO taken twice daily.
3. Zidovudine 300 mg PO twice daily + Lamivudine 150 mg PO twice daily + Efavirenz 600 mg PO taken once daily.

STATISTICAL METHODS:

The primary efficacy variables are the percentage of subjects with HIV-1 RNA levels <400 copies/mL and the percentage of subjects with HIV-1 RNA levels <50 copies/mL. A step down procedure will be used with percentage <400 tested first, and if non-inferiority is demonstrated then percentage <50 will be tested. The primary efficacy time point is 48 weeks. The primary efficacy variable will also be analyzed at 24 weeks and 96 weeks. For the primary analysis one-sided 98.75% confidence intervals for the difference in percentages between each of the UK-427,857 dose groups and the efavirenz control arm will be provided using the normal approximation. These will be adjusted based on the randomization strata. Percentage of subjects with HIV-1 RNA levels <400 copies/mL and percentage of subjects with HIV-1 RNA levels <50 copies/mL will also be analyzed using logistic regression including screening viral load level (randomization strata), geographic region and treatment arm as factors. The other main secondary efficacy variables will be change from baseline in viral load, time averaged difference in viral load, change from baseline in CD4 cell count, change from baseline in CD8 cell count, time to virological failure (analyzed using survival methods), association between baseline resistance and virological response and changes in genotype, phenotype and/or tropism in treatment failures after Week 4.

An interim analysis will be performed and the results reviewed by the DSMB.

01000004273272\1.0\Approved\24-Aug-2004 11:39

VISIT SCHEDULE:

Procedures	Screening (Day – 42 to -28)	Randomization (Day –7 to –4)	Baseline[a] Day 1	Week 2 [b]	Weeks 4, 8, 12, 16, 20, 32, 40, 60, 72, 84[b]	Weeks 24, 48, 96 or Early Termination [b,c]
Informed Consent and Eligibility Check	X					
Medical History			X			
Physical Exam/Vital Signs			X			X
Targeted Physical Exam/Vital Signs					X	
Waist/Hip Lipodystrophy Measurements			X			X
Adverse Events			X	X	X	X
Concomitant Medications			X	X	X	X
Chemistry, Hematology	X		X	X	X	X
Fasting Metabolic Assessment (total cholesterol, HDL/LDL, triglycerides, glucose, glycosylated hemoglobin)			X			X
12-lead Electrocardiogram			X			X
Orthostatic BP Monitoring	X		X[d]	X		X
PK Sampling[e]				X	X	X
Urinalysis			X			X
Hepatitis screen (B core Ab, sAg, sAb, C Ab)	X					
Hepatitis C Virus RNA[f]			X		X	X
CD4/CD8	X		X	X	X	X
Plasma HIV-1 RNA	X	X	X	X	X	X
Pregnancy Test[g]	X		X		X	X
Plasma/PBMC/Proviral DNA Storage[h]			X		X	X
Viral Resistance (Phenotype, Genotype)[i]	X				X[j]	X
Co-receptor tropism (Phenotype, Genotype[k])	X		X	X	X[l]	X
Host Genotyping			X			
Free T4, TSH			X			X
ACTG Symptom Distress Module			X		X [m]	X
CT Scan (Abdomen and Thigh) [n]			X			X
Dispense Study Medication			X	X[o]	X	X[p]
Assess Dosing Compliance				X	X	X

01000004273272\1.0\Approved\24-Aug-2004 11:39

a. Day 1, prior to dosing.
b. All visits must occur within ±4 days.
c. Subjects who discontinue study drug due to treatment failure or for other reasons must be followed per protocol until Week 96.
d. Patients with asymptomatic postural hypotension at the Baseline visit will be monitored for 4 hours following the first dose of study drug.
e. Two 5 ml PK samples are required at Weeks 2 and 48 and must be at least 30 minutes apart. One 5 ml PK sample required at other visits. Through Week 48 only.
f. If Hepatitis C antibody is positive at Screening Visit, to be performed at Baseline, Weeks 12, 24, 48 and 96 or Early Termination.
g. For Women of Child Bearing Potential. Serum pregnancy at Screening and Urine Tests at the following visits. A positive Urine test must be confirmed with a serum test.
h. Plasma aliquots (2 of 1 mL each) at all timepoints. PBMCs, and proviral DNA will be stored at Baseline and at Weeks 24 , 48 and 96 or upon treatment failure only.
i. Reverse transcriptase inhibitor resistance testing to determine eligibility and gp 160 sequencing.
j. Upon treatment failure only, as defined in the protocol.
k. Genotype (V3 loop alone or as part of gp 160 sequencing) at Baseline, Weeks 24, 48 and 96 and at treatment failure only.
l. Phenotype at these visits and upon treatment failure as defined in the protocol.
m. Week 12 only.
n. Performed in a subset (target n = 300) of patients from selected centers at Baseline and Week 96 or at Early Termination on or after Week 48.
o. Container from previous visit.
p. At Week 96 or Early Termination, medication will be dispensed to subjects who have completed 96 weeks of therapy and for whom it is medically appropriate to continue or begin therapy with UK-427,857. (See Section 5.6).

010000042732721.0\Approved\24-Aug-2004 11:39

TABLE OF CONTENTS

1. INTRODUCTION 10
1.1. Background 10
1.1.1. Data from Healthy volunteer Studies 11
1.1.2. Data from Patient Studies 12
1.2. Rationale 13
1.2.1. UK-427,857 in treatment-naïve patients: 13
1.2.2. Efavirenz in treatment-naïve patients: 14
1.2.3. Dose selection: 14
2. TRIAL OBJECTIVES 16
3. TRIAL DESIGN 17
4. SUBJECT SELECTION 19
4.1. Inclusion Criteria 20
4.2. Exclusion Criteria 20
4.3. Life Style Guidelines 22
5. TRIAL TREATMENTS 22
5.1. Allocation to Treatment 22
5.2. Breaking the Blind 23
5.3. Drug Supplies 23
5.3.1. Formulation and Packaging 24
5.3.2. Preparation and Dispensing 24
5.3.3. Administration 24
5.3.4. Compliance 24
5.4. Drug Storage and Drug Accountability 25
5.5. Concomitant Medication(s) 25
5.6. Rescue Therapy 26
6. TRIAL PROCEDURES 26
6.1. Screening Visit (Day –42 to Day –28) 27
6.2. Trial Period 27
6.2.1. Randomization Visit (Day -7 to Day -4) 27
6.2.2. Baseline (Day 1) Evaluation – *Prior to first dose* 27

0100000427327\1.0\Approved\24-Aug-2004 11:39

6.2.3. Week 2 Visit 28
6.2.4. On Study Evaluations (Week 4, 8, 12, 16, 20, 32, 40, 60, 72 and 84) 29
6.2.5. Week 24, 48 and 96 or Early Termination Visit 30
6.3. Population Pharmacokinetics 31
6.3.1. Population Pharmacokinetics: Dosing history 31
6.3.2. Population Pharmacokinetics: Meal times history 31
6.4. Drug Assay 31
6.5. Virology Testing 31
6.5.1. Viral load measurements 31
6.5.2. Virus tropism 32
6.5.3. Viral resistance 32
6.5.4. Virus susceptibility to UK-427,857 33
6.5.5. Blood Pressure, Heart Rate and ECG Monitoring 33
6.5.6. Host Genotyping 34
6.6. Subject Withdrawal 34
7. ASSESSMENTS 36
7.1. Efficacy Assessments 36
7.2. Safety Assessments 36
8. ADVERSE EVENT REPORTING 36
8.1. Adverse Events 37
8.2. Definition of an Adverse Event 37
8.3. Abnormal Laboratory Findings 38
8.3.1. Laboratory Tests will include: 38
8.3.2. Follow Up of Laboratory Test Abnormalities: 39
8.4. Serious Adverse Events 40
8.5. Hospitalization 40
8.6. Severity Assessment 41
8.7. Exposure In Utero 42
8.8. Discontinuations (See also Subject Withdrawal, Section 6.6) 43
8.9. Eliciting Adverse Event Information 43
8.10. Reporting Requirements (Serious and Nonserious) 43

0100000427327211.0\Approved\24-Aug-2004 11:39

8.11. Additional Safety Information ... 44
9. DATA ANALYSIS/STATISTICAL METHODS ... 44
9.1. Sample Size Determination ... 44
9.2. Efficacy Analysis ... 45
9.2.1. Analysis Populations ... 45
9.2.2. Analysis of Primary Endpoint ... 45
9.2.2.1. Primary Endpoint ... 45
9.2.2.2. Method of Analysis ... 46
9.2.3. Analysis of Secondary Endpoints ... 46
9.2.3.1. Secondary Endpoints ... 46
9.2.3.2. Method of Analysis ... 47
9.3. Analysis of Other Endpoints ... 49
9.3.1. Pharmacokinetic Sampling and Population PK ... 49
9.4. Safety Analysis ... 49
9.5. Interim Analysis ... 49
9.6. Data Monitoring Committee ... 50
10. QUALITY CONTROL AND QUALITY ASSURANCE ... 51
11. DATA HANDLING AND RECORD KEEPING ... 51
11.1. Case Report Forms / Electronic Data Record ... 51
11.2. Record Retention ... 52
12. ETHICS ... 52
12.1. Institutional Review Board (IRB)/Independent Ethics Committee (IEC) ... 52
12.2. Ethical Conduct of the Trial ... 52
12.3. Subject Information and Consent ... 52
13. SPONSOR DISCONTINUATION CRITERIA ... 53
14. PUBLICATION OF STUDY RESULTS ... 53
15. REFERENCES ... 54

0100000427327 2\1.0\Approved\24-Aug-2004 11:39

010000042732721\1.0\Approved\24-Aug-2004 11:39

1. INTRODUCTION

Over 40 million people worldwide are now infected with HIV with an estimated 5 million new infections in 2003.[4] At present the only means of slowing disease progression is to use a multi-drug combination strategy working against three HIV targets, protease, reverse transcriptase and gp-41. These agents reduce the rate of virus replication, while seeking to prevent the rapid emergence of resistant virus. While several of these combinations are successful, the side-effect profiles can reduce patient compliance. In addition, current drug combination therapies are unable to overcome the emergence of multi-drug resistant viruses. Thus there is a high medical need for better tolerated, conveniently administered agents to prevent the development and progression of AIDS in HIV infected individuals, reduce susceptibility to secondary infections, and return the patient to a normal lifestyle. Furthermore, in order to delay the development of resistance, the development of new classes of drugs, which work on different stages of the life cycle of the virus, is necessary.

An essential step in the replication cycle of HIV is attachment to both the CD4 receptor and one of the chemokine receptors (CCR5 in the case of primary infection and earlier stages of disease). Blocking of the CCR5 receptor prevents viral entry into the host cell and will therefore lead to a drop in viral load. This has been demonstrated in initial Phase IIa studies, with the CCR5 antagonists SCH-C and UK-427,857, and more recently with SCH-D. All compounds significantly reduced the viral load in HIV infected patients receiving monotherapy over 10-14 days.[5,6,7] These results confirmed genetic evidence from previous *in vitro* studies that indicated that blocking of the CCR5 receptor has a profound inhibitory effect on viral infection.

CCR5-tropic HIV-1 is known to predominate during early HIV-infection with the risk of harboring CXCR4-tropic virus (either with R5 virus or as the predominant viral species) increasing significantly over time. In studies A4001007 and A4001015 combined, which screened for co-receptor tropism phenotype in HIV-1 infected patients with CD4 counts greater than 250 cells/mm^3 and either naïve to therapy or off antiretroviral therapy for at least 8 weeks, the proportion of patients infected with CCR5-tropic HIV-1 was in excess of 90%.[8] Interim data from a retrospective study of two large antiretroviral-naïve cohorts (with no CD4 count restriction) show that the incidence of CCR5-tropic HIV-1 is approximately 85%.[9,10] Thus, CCR5 inhibition as a component of HAART, may provide a benefit for a sizable proportion of the treatment-naïve population.

1.1. Background

UK-427,857 is a small molecule antagonist of the CCR5 receptor with potent antiviral activity *in vitro* (geometric mean antiviral IC_{90} against a laboratory-adapted strain [BaL] of 5.57 nM, 95% CI: 3.98 - 7.78nM [2.85 ng/ml, 95% CI: 2.04-4.0 ng/ml]; and against a range of primary HIV-1 isolates [n=44] of 2.03 nM, 95% CI: 1.75-2.36 nM [1.04, 95% CI: 0.9-1.22 ng/ml]).

Further details of pre-clinical and toxicological data may be found in the Investigator's Brochure.

1.1.1. Data from Healthy volunteer Studies

To date more than 400 healthy volunteers have received UK-427,857. Single oral doses up to 900 mg and multiple oral doses up to 300 mg BID for 28 days were well tolerated.

The most common Adverse Events (AEs) reported following UK-427,857 dosing have been asthenia, headache, flatulence and nausea. Postural hypotension is potentially the dose limiting adverse event. No Serious Adverse Events (SAEs) have been reported and most AEs were judged to be mild to moderate in severity.

In the single dose study (A4001001) four out of nine subjects who were administered a single 1200 mg dose of UK-427,857 experienced postural hypotension. Clinically significant postural hypotension caused dosing to be halted for Cohort 3 of the multiple dose study (A4001002) with 2/9 subjects on 600 mg QD and 1/3 on placebo. Dosing was repeated at 600 mg QD in an additional group of subjects (Cohort 5) and was well tolerated, with 1 subject experiencing mild postural hypotension. Hence the maximum tolerated multiple dose remains undefined. Single and repeated dosing with UK-427,857 had no apparent effects on supine systolic or diastolic blood pressure, although there was evidence of a treatment-related increase in supine pulse rate after a single 1200 mg dose (mean increase from baseline of 9 bpm at 1 hour post- dose). There were dose-related reductions in standing systolic blood pressure at doses of 300 mg BID and above and for standing diastolic blood pressure at 600 mg QD.

Analysis of the single dose, adequately powered QT study with active control (A4001016) has shown that the mean difference in QTcI from placebo for all primary endpoints was less than 4 msec for all doses of UK-427,857 (100 mg, 300 mg and 900 mg). Mean differences from placebo in QTcI for UK-427,857 were not clinically significant for single doses up to and including 900 mg.

UK-427,857 absorption is rapid but variable after oral dosing and C_{max} typically occurs 0.5–4h after dosing. Assessment of UK-427,857 over the dose range 50 to 600 mg confirmed that the pharmacokinetics of UK-427,857 are not dose proportional. It is estimated that a doubling in dose will lead to a 2.3-fold increase (95% CI; 2.2, 2.4-fold) in mean AUC over this dose range. UK-427,857 has biphasic elimination with a measured terminal half-life of 9 to 14 hours following single doses.

UK-427,857 is a CYP3A4 substrate. Hence its concentrations are increased by potent CYP3A4 inhibitors (such as ketoconazole, saquinavir and ritonavir) and decreased by CYP3A4 inducers (such as rifampin and efavirenz).

From pre-clinical data, UK-427,857 is not predicted to affect the pharmacokinetics of other drugs that are metabolized by any of the major P450 enzymes. Clinical studies have demonstrated that UK-427,857 does not lead to clinically significant changes in plasma concentrations of oral contraceptives (ethinyloestradiol and levonorgestrel) or CYP3A4 substrates such as midazolam.

01000004273272\1.0\Approved\24-Aug-2004 11:39

1.1.2. Data from Patient Studies

In two short-term monotherapy studies, A4001007 and A4001015, seventy-nine asymptomatic CCR5 tropic HIV-1 infected patients with CD4 count >250 cells/mm^3 and plasma viral load >5000 copies/ml received UK-427,857 (in total daily doses ranging from 25 mg to 600 mg) or placebo for 10 days. All doses were given in the fasted state or under food restrictions, apart from 150 mg BID, where both a fed and fasted dose were given. Mean viral load reductions of 0.43, 1.13, and 1.35 $\log_{10}$ were seen for doses of 25, 100 and 300 mg QD, respectively. BID doses of 50, 100, 150 and 300 mg resulted in viral load reductions of 0.66, 1.42, 1.45, and 1.6 $\log_{10}$, respectively. Patients receiving 150 mg BID fed had a viral load decline of 1.34 $\log_{10}$, indicated a negligible impact of food on viral load reduction. UK-427,857, was safe and well-tolerated in this patient population. The AE profile was similar to that seen for healthy volunteers.

There was a change in virus tropism in two individuals treated with UK-427,857, both in study A4001015. One subject received UK-427,857 100 mg QD and had a viral load decline of 0.710 log10 from baseline. Analysis of virus tropism on Day 11 demonstrated the presence of virus with the ability to enter cells expressing the CXCR4 receptor. By Day 40 the virus population had reverted to CCR5 only usage. Screening of multiple clones obtained from the virus population at the different time points revealed the presence of a small number of clones (2 out of 97 viable clones) with the ability to enter cells expressing CXCR4 at baseline. By Day 11, 40 of 68 clones could enter CXCR4 expressing cells, while only 1 of 91 could enter into CXCR4 expressing cells by Day 40. These data are consistent with the presence of a small population (<5%) with the ability to enter into cells expressing the CXCR4 receptor at baseline with a relative increase on Day 11, reverting back to baseline following withdrawal of UK-427,857.

The second subject received UK-427,857 100 mg QD and had a viral load decrease of 1.256 log10 from baseline. In this subject R5X4 virus emerged by Day 11 and has been detectable at all time points since. A detailed clonal analysis of the virus population at the different time points was performed. Of 118 viable clones evaluated at baseline, none had the ability to use CXCR4 for entry. At Day 11, 21 of 52 clones could use the CXCR4 receptor for entry, while 17 of 63 could use CXCR4 at Day 40. To evaluate the possibility that the R5X4 tropic virus present at Days 11 and 40 could have arisen from the CCR5 population present at baseline, gp160 sequencing and phylogenetic analyses of 12 randomly selected clones from each time point were performed. Analysis of this data revealed that the R5X4 viruses present at Days 11 and 40 were closely related, but genetically distinct from the R5 population. Direct sequence comparisons indicated that there were 104 nucleotide differences between a representative clone from the R5X4 population and the most closely related R5 virus. At the amino acid level there were 55 differences, including conserved amino acids, using the same comparison. It is, therefore, very unlikely that the R5X4 viruses could have originated from the R5 population during the 10 day period of dosing with the CCR5 antagonist. It is more likely, therefore, that the R5X4 virus has been present prior to dosing with UK-427,857, either at a very low prevalence (<1%) in the peripheral circulation, or as archived virus in a tissue compartment. The large CD4 T-cell increase (173 cells/mm3 over 10 days) in this patient could indicate

090000042732721.0\Approved\24-Aug-2004 11:39

redistribution of cells from central compartments to the periphery and may have resulted in archived virus entering the circulation.

Further follow-up samples taken from this patient at days 203, 251 and 308 demonstrated that the R5X4 virus was still present in the peripheral circulation, with emergence of X4 variants over the later sampling points. Over the >9 months period since the patient received UK-427,857, his viral load has been variable but remains within the range of his pre-study values (viral load at randomization visit was 80471 copies/ml). However, there has been a gradual decline in his CD4 count (318 cells/mm3 on Day 308). This is an approximately 50% decline from his immediate pre-study CD4 count and is also 25% lower than his lowest count (421 cells/mm3) recorded over the past 6 years of follow-up. While the precise role of R5 and X4 viruses in HIV pathogenesis is not known, in light of the evolving data, the possibility that UK-427,857 selected the X4 species over R5 and that this may be causally related to the decline in CD4 cell counts cannot be excluded. The patient remains clinically well and has not initiated HAART at this time.

Further information can be obtained from the current version of the Investigator's Brochure.

1.2. Rationale

010000042732721.0\Approved\24-Aug-2004 11:39

1.2.1. UK-427,857 in treatment-naïve patients:

The rationale for studying UK-427,857 for the treatment of HIV-1 infection is based on the fact that persons who are heterozygous for the Δ32 mutant CCR5 allele (ie, fewer functional CCR5 receptors) have lower serum viral loads, a better response to highly active antiretroviral therapy (HAART) and delayed progression to AIDS or death. Treatment-naïve patients are arguably the most appropriate patient population to study, since the chemokine receptor CCR5 is the primary co-receptor for nonsyncytium inducing HIV-1, which predominates during the early stages of HIV-1 infection. While the number of approved antiretroviral agents has increased substantially over the past decade, there remain fundamental problems with members of all four major classes including low genetic barrier to resistance (non-nucleoside reverse transcriptase inhibitors), cross-class resistance (all), mitochondrial toxicity (nucleoside reverse transcriptase inhibitors), lipodystrophy (protease inhibitors) and mode of administration (fusion inhibitors).

Complicating matters even further, the prevalence of transmitted virus resistant to existing antiretroviral agents is increasing. In a retrospective study of North American patients who were recently infected with HIV-1, the percentage of those with at least one drug-resistance mutation increased from 8% in 1995-1998 to 22.7% in 1999-2000.[11] Results of the CATCH study revealed the overall rate of primary genotypic resistance in Europe to be close to 10%, with nearly 2% of all isolates exhibiting multi-class resistance.[12] Two recent cases of resistant HIV-1 transmission were identified where resistant virus persisted for three years without reversion to wild type. This was associated with more rapid disease progression.[13] Not surprisingly, the recent IAS-USA guidelines recommends resistance testing in patients who have been infected for 2 years or more.[14] As resistance to current classes continues to climb, there will be a need for newer therapeutic options with novel mechanisms of action that can effectively treat patients with transmitted drug-resistant HIV-1 and that are safe and well tolerated.

1.2.2. Efavirenz in treatment-naïve patients:

A regimen consisting of a nonnucleoside reverse transcriptase inhibitor (NNRTI) plus two nucleoside reverse transcriptase inhibitors (NRTIs) is perhaps the preferred regimen for the treatment of established human immunodeficiency virus (HIV) infection in antiretroviral-naïve patients. The recent Department of Health and Human Services (DHHS) Guidelines recommends efavirenz plus other agents (two nucleosides or nucleoside/nucleotide combination) as the preferred non-nucleoside-based starting regimen.[15] An efavirenz-based regimen has been shown to be as or more effective than protease-inhibitor (PI) based regimens, with or without ritonavir-boosting, in multiple studies.[16,17,18,19] It has the advantage of sparing the use of protease inhibitors with their associated long-term toxicities (eg, lipid abnormalities, lipodystrophy).

Despite the popularity of efavirenz-based therapy, however, there are several disadvantages including resistance conferred (to the entire NNRTI class) by a single or limited number of mutations (K103N) as well as safety issues. Side effects include rash (15 to 27%; Stevens-Johnson syndrome in 0.05%), hyperlipidemia, increased aminotransferase levels (levels >5x ULN in 2-8%) and CNS toxicity in up to 52% of patients.[20] Although it is generally believed that the latter can be "managed" in clinical practice, a recent study indicated that efavirenz-induced CNS toxicity might be underestimated. Thirteen percent (13%) of treatment-naïve patients who stopped efavirenz within the first 2 years did so due to CNS toxicity. Discontinuation rates did not diminish over time, suggesting that unmanageable CNS toxicity may emerge or worsen beyond the first months of therapy.[21] In addition, recent data suggests that transmission of K103N resistant HIV-1 may persist for 3 years or more without reversion to wild-type.[22] Furthermore, low-level transmission of K103N resistant HIV-1 may escape routine genotypic detection and lead to failure of an efavirenz-based regimen with persistence of these NNRTI-resistant variants for up to 5 years or more.[23] Lastly, efavirenz is a category C drug and should not be used during pregnancy due to birth defects seen in cynomolgus monkeys.

0100000427327 2\1.0\Approved\24-Aug-2004 11:39

1.2.3. Dose selection:

The rationale for dose selection is to maximize the benefit: risk ratio for UK-427,857 in treatment-naïve patients. The dose selection is based on 10 day monotherapy data, PK/PD modeling, clinical trial simulations, pharmacokinetics, drug-drug interaction studies, preclinical serial passage resistance studies and a safety database of over 400 subjects followed for up to 4 weeks.

Consideration of the integrated safety and toleration data generated in the Phase I healthy volunteer studies and Phase 2a monotherapy studies has enabled the optimal balance between the efficacy data discussed above and the potential safety and toleration burden. The major factor identified thus far that determines the maximum tolerated/safe clinical dose is postural hypotension. However, no safety data are available for long-term combination therapy in HIV infected patients at this stage and therefore the assumption is that short-term monotherapy data are predictive of that situation.

As previously noted, 2 studies (A4001007 and A4001015), have been performed in treatment naïve patients, investigating the anti-viral effects, pharmacokinetics, safety and toleration of various doses and regimens of UK-427,857. The conclusions from these studies are that anti-viral effect appears to be related to daily dose/exposure, such that similar anti-viral effects are seen for total daily doses of above 200 mg, administered as a once or twice a day regimen. In addition the anti-viral effects of a 150 mg BID dose do not appear to be affected by co-administration with food. Maximal anti-viral effects appear to occur at doses of 100 mg BID (200 mg/day) and above. The anti-viral effects of these doses are in line with those seen with short term monotherapy for other anti-retroviral agents, which have subsequently shown robust long term anti-viral effects in combination with other anti-retroviral agents in treatment naïve patients.

The anti-viral data from these short term monotherapy studies have been incorporated into a fully integrated drug-disease model which has subsequently been used to simulate the outcome of Phase 2b/3 trials in treatment naïve patients receiving zidovudine/lamivudine in combination with UK-427,857. Given that there is no long-term combination data available for any CCR5 inhibitor, a number of assumptions around resistance, compliance, adherence and baseline viral load have been made. Together, the monotherapy and clinical trial simulation (CTS) data have enabled rationale selection of doses to be taken into the Phase 2b/3 studies based on anti-viral efficacy.

A model was used to simulate the UK-427,857 (plus zidovudine/lamivudine) dose response curve for the success rate measured as the proportion of patients with <400 copies/mL at Week 24 and Week 48, assuming no UK-427,857 resistance. The proportion of patients with <400 HIV-1 RNA copies/mL increased with increasing dose. The results from the simulations suggested that a 71% success rate at 48 weeks in an intention-to-treat population is predicted for 300 mg BID in treatment naïve patients with a baseline viral load of 4.8 $\log_{10}$. For 300 mg QD, a 62% success rate is predicted at 48 weeks, however a much higher success rate is predicted at Week 24 (approximately 77%). The failure rate post 24 weeks is primarily driven by predicted emergence of resistance to zidovudine/lamivudine rather than to UK-427,857.

Taken together, the efficacy data and clinical trial modeling for treatment-naïve patients, demonstrate that doses of 200 mg to 600 mg daily have similar effects on viral load. Efficacy appears to be driven by daily dose and is independent of the regimen by which it is administered at daily doses of above 100 mg. A daily dose of 300 mg BID (600 mg daily) is likely to be towards the top of the dose response curve, while a daily dose of 150 mg BID (300 mg daily) is also likely to provide a good response. These doses produced a short-term decline in HIV-1 RNA comparable to other agents, which are considered to be acceptable “anchor drugs” in a typical HAART regimen. Additionally, since it has been difficult to demonstrate a dose-response based on efficacy in Phase 2b studies with other antiretroviral agents, a 50% reduction in the total daily dose is likely to result in the best opportunity to achieve this. It is also clear that the dose limiting adverse event (postural hypotension) is related to plasma levels of UK-427,857, with the incidence being the greatest around the C_{max} of the doses at which it occurs. Given the high medical need to simplify dose regimens and enhance adherence, as well as the potency of UK-427,857 when given as a 300 mg once-daily dose compared with other agents

010000042732721.0\Approved\24-Aug-2004 11:39

(eg, atazanavir), we propose to study 300 mg QD and 300 mg BID in this study. These doses will maximize the antiviral effects while minimizing the chances of postural hypotension and other potential safety and toleration issues.

2. TRIAL OBJECTIVES

Primary Objective:

To assess whether the antiviral activity (ie, fewer than 400/50 HIV-1 RNA copies per mL of plasma at Week 48), of each of two doses of UK-427,857 in combination with zidovudine/lamivudine is non-inferior to a reference regimen of efavirenz plus zidovudine/lamivudine in antiretroviral-naïve, CCR5-tropic HIV-1 infected subjects.

Secondary Objectives:

1. To assess whether the antiviral activity (ie, fewer than 400/50 HIV-1 RNA copies per mL of plasma at Week 24), of each of two doses of UK-427,857 in combination with zidovudine/lamivudine is non-inferior to a reference regimen of efavirenz plus zidovudine/lamivudine in antiretroviral-naïve, CCR5-tropic HIV-1 infected subjects.
2. To assess whether the antiviral activity (ie, fewer than 400/50 HIV-1 RNA copies per mL of plasma at Week 96), of each of two doses of UK-427,857 in combination with zidovudine/lamivudine is non-inferior to a reference regimen of efavirenz plus zidovudine/lamivudine in antiretroviral-naïve, CCR5-tropic HIV-1 infected subjects.
3. To compare the time to loss of virological response through Weeks 48 and 96 for each of the two UK-427,857 regimens versus the efavirenz regimen.
4. To compare the reduction of plasma $\log_{10}$ HIV-1 RNA from baseline through Weeks 24, 48 and 96 for each of the two UK-427,857 regimens versus the efavirenz regimen.
5. To compare the differences in the magnitude of changes in CD4 cell counts from baseline through Weeks 24, 48 and 96 for each of the two UK-427,857 regimens versus the efavirenz regimen.
6. To compare the differences in the magnitude of changes in CD8 cell counts from baseline through Weeks 24, 48 and 96 for each of the two UK-427,857 regimens versus the efavirenz regimen.
7. To compare the Time-Averaged Difference (TAD) in $\log_{10}$ HIV-1 RNA at Weeks 24, 48 and 96 for each of the two UK-427,857 regimens versus the efavirenz regimen.
8. To assess HIV-1 genotype and phenotype at baseline and at the time of failure (patients with HIV-1 RNA greater than 400 copies/mL at any visit after Week 4, or other reasons for treatment failure listed at end of Section 3).
9. To assess HIV-1 tropism at baseline and at the time of failure (patients with HIV-1 RNA greater than 400 copies/mL at any visit after Week 4, or other reasons for treatment failure listed at end of Section 3).

010000042732721\1.0\Approved\24-Aug-2004 11:39

10. To assess the association between baseline resistance and virological response.
11. To compare the safety and tolerability of each of the two UK-427,857 regimens versus the efavirenz regimen.

3. TRIAL DESIGN

This is a 96-week, multi-national, multi-center, double blind, randomized (1:1:1), comparative, non-inferiority Phase 2b/3 hybrid (run-in) study designed to compare the safety and antiviral activity of UK-427,857 at two different doses versus efavirenz, each in combination with zidovudine/lamivudine. This study is planned to enroll a total of 1071 subjects. Patients eligible for enrollment will be males or females at least 16 years of age, with documented HIV-1 infection and more than 2000 copies of HIV-1 RNA per milliliter of plasma (measured by Roche Amplicor HIV-1 Monitor, version 1.5) and CCR5 co-receptor viral tropism as determined by the PhenoSense™ HIV entry assay (ViroLogic, Inc, South San Francisco, CA). Major exclusion criteria would include more than 14 days of treatment with any antiretroviral therapy, treatment for an opportunistic infection within 30 days prior to screening, suspected primary HIV-1 infection and/or evidence of X4- or dual-tropic virus or repeated assay failure at baseline.

All subjects will undergo genotypic and phenotypic testing for the presence of non CCR5-tropic HIV-1 and for resistance to reverse transcriptase inhibitors to determine eligibility. Screening genotype and phenotype results will be available approximately 3 to 5 weeks after receipt of samples. The percentage of patients with HIV-1 RNA less than 400 copies/mL will be assessed at 24, 48 and 96 weeks or at the time of discontinuation from the study. All subjects who receive at least 1 dose of study medication will be assessed for safety. The Investigators, patients and Pfizer personnel directly involved in the conduct of the study will remain blinded to this analysis. The study will enroll over approximately a 18-month period (5 months run-in, 13 months Phase 3) with 96 weeks of treatment. The primary efficacy endpoint will be assessed after 48 weeks of treatment. As part of this clinical study a blood sample will be taken for non-anonymized pharmacogenetic analysis.

0100000427327 2\1.0\Approved\24-Aug-2004 11:39

A Data Safety Monitoring Board (DSMB) will review the results following treatment of 75 to 100 patients for at least 8 weeks. No formal statistical tests will be performed. If the DSMB feels that either of the doses of UK-427,857 show substantial evidence of harm to patients, then the DSMB will recommend ending recruitment to this treatment arm. In addition, Pfizer Worldwide Safety and Risk Management will conduct regular blinded reviews of serious adverse events (weekly) and adverse clinical and laboratory events (monthly) with particular focus on new infections, liver enzyme abnormalities and postural hypotension.

Two hundred and four patients will be treated for 16 weeks during the run-in phase. A formal interim efficacy analysis is planned to test non-inferiority of each UK-427,857 regimen in Time-Averaged Difference (TAD) in viral load versus the efavirenz regimen. Assuming a one-sided significance level of 0.025, 95% power, a non-inferiority margin of 0.5 and common standard deviation of 0.8, 68 patients per treatment group will be required. With 68 patients per group, there would also be 80% power to detect a difference between the two UK-427,857 regimens in time-averaged drop in viral load of 0.387 $\log_{10}$. Assuming a 90% response rate

(percentage with HIV-1 RNA less than 400 copies/mL) there is over 95% power to show non-inferiority with a non-inferiority margin of -20%. The DSMB will consider whether a UK-427,857 dose not showing non-inferiority to efavirenz should be dropped at this stage. The study will not be stopped to make a claim for efficacy for UK-427,857 at the interim analysis. The Investigators, patients and Pfizer personnel directly involved in the conduct of the study will also remain blinded to this analysis.

This study will evaluate two doses of UK-427,857 (300 mg once daily and 300 mg twice daily) versus efavirenz (600 mg once daily) each taken in combination with zidovudine/lamivudine (300 mg/150 mg twice daily). Subjects randomized to the UK-427,857 300 mg QD arm will receive UK-427,857 placebo drug in the morning and UK-427,857 active drug plus efavirenz placebo drug in the evening. Subjects randomized to the UK-427,857 300 mg BID arm will receive UK-427,857 active drug in the morning and UK-427,857 active drug plus efavirenz placebo drug in the evening. Subjects randomized to the efavirenz arm will receive UK-427,857 placebo drug in the morning and in the evening and efavirenz active drug in the evening. UK-427,857 and efavirenz may be taken with or without food. Zidovudine/lamivudine will be provided in open-label fashion and should be taken twice daily, with or without regard to food, as per the manufacturer's product labeling. Randomization will be stratified by screening HIV-1 RNA levels (<100,000 versus ≥100,000 copies/mL) and by geographic location (ie, Northern Hemisphere/Southern Hemisphere).

Subjects will be randomly assigned to one of the following treatment groups:

- Zidovudine 300 mg PO twice daily + Lamivudine 150 mg PO twice daily + UK-427,857 300 mg PO taken once daily
- Zidovudine 300 mg PO twice daily + Lamivudine 150 mg PO twice daily + UK-427,857 300 mg PO taken twice daily
- Zidovudine 300 mg PO twice daily + Lamivudine 150 mg PO twice daily + Efavirenz 600 mg PO taken once daily

Subjects will remain on their assigned therapy for 96 weeks, unless the subject is discontinued early for protocol-defined treatment failure or other reasons such as adverse event, loss to follow-up, withdrawal of consent or death. These subjects will be required to remain in the studies on their new regimen and be followed as defined in the protocol. Subjects experiencing toxicity, but not failure, attributed to zidovudine/lamivudine would be able to substitute an alternative nucleoside analogue in consultation with the medical monitor. The primary efficacy analysis will be conducted following 48 weeks of therapy. Co-receptor tropism testing will be performed in all patients with HIV-1 RNA greater than 500 copies/mL at Weeks 2, 4, and then every 4 weeks to Week 24, every 8 weeks to Week 48 and every 12 weeks to Week 96, as well as in all patients who meet any of the criteria for treatment failure.

010000042732721.0\Approved\24-Aug-2004 11:39

Treatment failure is defined as meeting any one of the following virological endpoints:

- An increase to at least 3 times the baseline (mean of all 3 values before start of dosing) plasma HIV-1 RNA level at the Week 2 visit or thereafter (confirmed by a second measurement taken no more than 14 days after the first measurement);
- Failure to achieve HIV-1 RNA <400 copies/mL by Week 24;
- HIV-1 RNA <1.0 $\log_{10}$ decrease from baseline (mean of all 3 values before start of dosing) at Week 4 or thereafter (confirmed by a second measurement taken no more than 14 days after the first measurement); or
- An increase in HIV-1 RNA to detectable levels (≥1,000 copies/mL on 2 consecutive measurements) in subjects previously confirmed to have undetectable levels of <400 copies/mL (confirmed by a second measurement taken no more than 14 days after the first measurement).

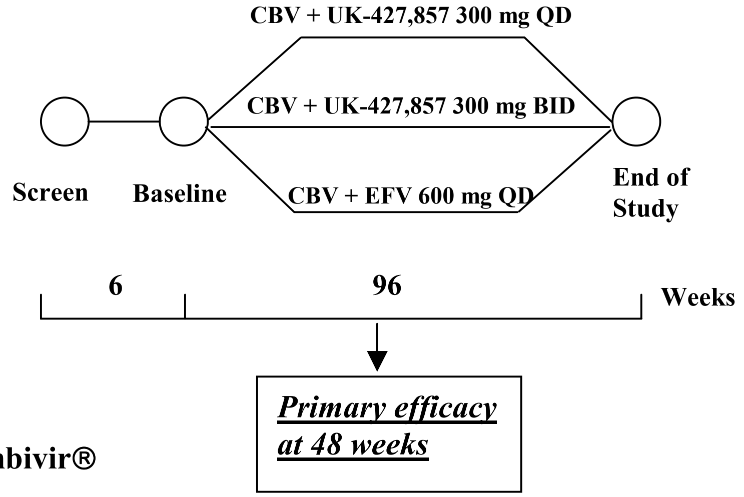

CBV = Combivir®

4. SUBJECT SELECTION

The target population for this study is HIV-1 infected patients over the age of 16, with HIV-1 RNA ≥2,000 copies/mL, CCR5-tropic virus as measured by PhenoSense™ viral tropism assay, HIV-1 susceptible to efavirenz, zidovudine and lamivudine, no more than 14 days of previous antiretroviral therapy and no active or recent (previous 30 days) opportunistic infection and no suspected primary HIV-1 infection.

This clinical trial can fulfill its objectives only if appropriate subjects are enrolled. The following eligibility criteria are designed to select subjects for whom protocol treatment is considered appropriate. All relevant medical and non-medical conditions should be taken into consideration when deciding whether this protocol is suitable for a particular subject.

0100000427327 2\1.0\Approved\24-Aug-2004 11:39

4.1. Inclusion Criteria

Subjects must meet all of the following inclusion criteria to be eligible for enrollment into the trial:

1. Provide a signed and dated written informed consent document indicating that the subject (or a legally acceptable representative) has been informed of all pertinent aspects of the trial.
2. Men or women at least 16 years of age (or minimum age as determined by local regulatory authorities or as legal requirements dictate) available for a follow-up period of at least 48 weeks.
3. HIV-1 RNA viral load of ≥2,000 copies/mL measured by Roche Amplicor HIV-1 Monitor (version 1.5) at the screening visit.
4. A negative urine pregnancy test at the baseline visit, prior to receiving the first dose of study medication, for Women of Child Bearing Potential (WOCBP).

   NOTE: WOCBP include any female who has experienced menarche and who has not undergone successful surgical sterilization or is not post-menopausal (ie, no menstrual periods for at least 2 years). Even women who are using oral, implanted or injectable contraceptive hormones or mechanical products (intrauterine devices; barrier methods: eg, condom or diaphragm with spermicide) to prevent pregnancy, who are practicing abstinence, or who have a partner that is sterile (eg, vasectomy), should be considered to be of child bearing potential.

5. Effective barrier contraception for WOCBP and males. In addition, WOCBP must use another acceptable method of contraception for the duration of the study. Acceptable contraception includes, but is not limited to, oral, implanted or injectable hormone therapy and intrauterine devices.

010000042732721.0\Approved\24-Aug-2004 11:39

4.2. Exclusion Criteria

Subjects presenting with any of the following will not be included in the trial:

1. Suspected or documented active, untreated HIV-1 related opportunistic infection (OI) or other condition requiring acute therapy (eg, hepatitis C virus infection) at the time of randomization [patients on a stable (>1 month) secondary OI prophylaxis regimen or chronic treatment (eg, for hepatitis C virus infection) are eligible for the study; patients on a primary OI prophylaxis regimen of any duration are also eligible for the study].
2. Treatment for an active opportunistic infection, or unexplained temperature >38.5°C for 7 consecutive days, within 30 days prior to randomization.
3. Prior treatment with efavirenz, zidovudine or lamivudine or with any other antiretroviral therapy for more than 14 days at any time.
4. Active alcohol or substance abuse sufficient, in the Investigator's judgment, to prevent adherence to study medication and/or follow-up.

5. Lactating women, or planned pregnancy during the trial period.
6. Suspected primary (acute) HIV-1 infection.
7. Previous therapy with a potentially myelosuppressive, neurotoxic, hepatotoxic and/or cytotoxic agent within 30 days prior to randomization or the expected need for such therapy during the study period.
8. Malignancy requiring parenteral chemotherapy that must be continued for the duration of the trial.
9. Documented or suspected acute hepatitis or pancreatitis within 30 days prior to randomization.
10. Renal insufficiency defined as a serum creatinine greater than 3 times the upper limit of normal.
11. Total bilirubin greater than 2 times the upper limit of normal.
12. AST and/or ALT greater than 3 times the upper limit of normal.
13. Cirrhosis of the liver.
14. Absolute neutrophil count ≤750 cells/mm^3.
15. Platelet count ≤50,000 cells/mm^3.
16. Hemoglobin ≤7 g/dL.
17. Clinically significant malabsorption syndrome (eg, ≥6 loose stools per day for at least 7 consecutive days) within 30 days prior to randomization.
18. Inability to tolerate oral medication.
19. Myocardial infarction, symptomatic angina or intermittent claudication within the previous 6 months.
20. Angiographically documented three-vessel coronary artery disease or left main stem disease.
21. Angiographically documented stenosis of >90% in the left anterior descending artery or a dominant right coronary or circumflex artery.
22. Congestive heart failure New York Heart Association Class III/IV.
23. History of a transient ischemic attack or cerebrovascular accident.
24. Documented carotid artery lesion of >70% or a carotid bruit (unless proven by ultrasound not to be due to carotid vascular disease).
25. Symptomatic postural hypotension (ie, systolic BP drop >20 mm Hg, diastolic BP drop >10 mm Hg and/or drop in systolic BP to <90 mm Hg) or postural hypotension (with or without symptoms) in a patient with hypertension being treated with one or more antihypertensive agents.
26. Concomitant therapy with other investigational agents.

01000004273272\1.0\Approved\24-Aug-2004 11:39

27. Contraindicated medications being taken by the subject at the time of randomization that must be continued during the study period, including immunomodulators (for the treatment of HIV-1 infection; interferon for the ongoing treatment of hepatitis C infection is permitted), ketoconazole, itraconazole, miconazole, clotrimazole, troleandomycin, nefazadone, clarithromycin, rifampin and rifabutin.
28. One or more nucleoside associated mutations conferring resistance to zidovudine including M41L, D67N, K70R, L210W, T215Y/F, K219Q/E/N, or phenotypic resistance to zidovudine.
29. Multi-nucleoside resistant genotype including the Q151M complex and codon 67-69 inserts.
30. One or more mutations conferring resistance to lamivudine including M184V/I, E44D, V118I, or phenotypic resistance to lamivudine.
31. One or more mutations responsible for efavirenz resistance including K103N, Y181C/I, Y188C/L/H, G190A/S, V106A, L100I, A98G, K101E, V108I, P225H, M230L or phenotypic resistance to efavirenz.
32. X4-or dual/mixed-tropic virus detected by the PhenoSense™ viral entry assay or repeated assay failure.
33. Any other clinical condition that, in the Investigator's judgment, would potentially compromise study compliance or the ability to evaluate safety/efficacy.

0100000427327 2\1.0\Approved\24-Aug-2004 11:39

4.3. Life Style Guidelines

Women of childbearing potential [any female who has experienced menarche and who has not undergone successful surgical sterilization or is not post-menopausal (ie, no menstrual periods for at least 2 years) must agree to avoid pregnancy during the study. WOCBP must use an acceptable method of contraception for the duration of the study and will be given advice on the correct use of contraceptives. Acceptable contraception includes, but is not limited to, oral, implanted (eg Norplant) or injectable hormone therapy (eg, Depo Provera) and intrauterine devices. In addition both males and females must agree to use a barrier method of contraception (eg, condom or diaphragm with spermicide) for the duration of the study.

5. TRIAL TREATMENTS

5.1. Allocation to Treatment

Eligible subjects who have provided written informed consent will be randomized into one of the three treatment groups in a 1:1:1 ratio (UK-427,857 300 mg QD : UK-427,857 300 mg BID : Efavirenz 600 mg QD). Subjects will be stratified at the time of randomization by screening HIV-1 RNA (<100,000 or ≥100,000 copies/mL) and by geographic location (Northern vs. Southern Hemisphere). Subjects will be randomized according to a computer generated pseudo-random code using the method of permutated blocks, balanced within each randomization strata (screening HIV-1 RNA ≥100,000 and Northern Hemisphere, screening HIV-1 RNA ≥100,000 and Southern Hemisphere, etc).

Randomization numbers will be assigned by a central web/telephone computer-based telerandomization system. The randomization call must be made at the Randomization Visit to allow for medication to be shipped to the site. This system will dispense medication by assigning appropriate container numbers to subjects based on their assigned treatment groups. Subject numbers will not appear on study medication containers, only container numbers will appear.

Only authorized site personnel will be granted access to the central randomization system. The following information will be required to randomize a subject:

- Site User ID (identification) and PIN (personal identification number), which are assigned to the investigational site by the randomization system;
- Protocol number;
- Subject's date of birth;
- Subject's gender.

The randomization system will generate a randomization number and a Single-Subject Identifier, which must be used on all documentation and correspondence. The Single-Subject Identifier will consist of the center number and a four-digit extension and will be assigned to all patients that sign a Patient Informed Consent document.

It is the responsibility of the Principal Investigator to ensure that the subject is eligible for the study before requesting randomization.

0100000427327\1.0\Approved\24-Aug-2004 11:39

5.2. Breaking the Blind

The UK-427,857 and efavirenz components of the three treatment regimens will be administered in double-blind, double-dummy fashion; UK-427,857 placebo and efavirenz placebo will be indistinguishable from the active study drug. The Investigator, study staff, subject and sponsor will be kept unaware of the treatment assignments until all outcome assessments (efficacy and safety) have been completed and the database has been locked. Zidovudine/lamivudine will be administered in open-label fashion.

At the initiation of the study, the study site will be instructed on the method for blind breaking. The method will be either a manual or electronic process. Blinding codes should only be broken in emergency situations for reasons of subject safety. The Investigator should contact Pfizer before breaking the blind. When the blinding code is broken, the reason must be fully documented and entered on the case report form.

In the event that an emergency break blind is required, authorized/approved randomization system users will have the ability to retrieve subject treatment groups through the randomization system.

5.3. Drug Supplies

- Drug supplies will be provided to the study sites as pre-packaged containers.

- Pfizer Global Research and Development will supply all study drugs relating to the study.
- Route of administration for all drug supplies is oral.

5.3.1. Formulation and Packaging

UK-427,857 tablets will be supplied as 150 mg dosage units. Matching placebo for UK-427,857 will also be supplied. UK-427,857 and placebo tablets will be presented in bottles.

Efavirenz (Sustiva ™) will be presented as 600 mg tablets. A matching placebo tablet will also be provided. Efavirenz 600 mg tablets and matching placebo will be presented in monthly containers.

UK-427,857 and efavirenz tablets will be supplied in a double-blind, double-dummy format.

Zidovudine 300 mg / Lamivudine 150 mg (Combivir™) tablets will be supplied as open label product, in equivalent packaging to the commercial packs.

5.3.2. Preparation and Dispensing

For each month of treatment (as defined by randomization), subjects will receive the following containers:

- 1 - Combivir™ tablets
- 2 - UK-427,857 (150 mg) and / or placebo tablets
- 1 - efavirenz 600 mg or placebo tablets

The quantities of study drugs dispensed per visit will be defined by the date of the next study visit, and will be managed via a telerandomization system.

01000004273272\1.0\Approved\24-Aug-2004 11:39

5.3.3. Administration

Study drug (UK-427,857 or efavirenz), as well as zidovudine/lamivudine, may be taken with or without food. The patient should only take missed doses if it is not within six hours prior to the planned next dose.

5.3.4. Compliance

Subjects will bring unused active drug/placebo tablets, as well as open-label zidovudine/lamivudine, to each study visit. The number of tablets will be counted and, if more than expected, subjects will be asked to account for missed doses. The Investigator and medical monitor will also evaluate subject compliance with the study regimen based on information such as plasma HIV-1 RNA levels. Potential reasons for non-compliance with UK-427,857 dosing (ie, AEs, lost medication) will be followed up by the study site personnel and strategies to improve dosing compliance will be explored.

5.4. Drug Storage and Drug Accountability

The Investigator, or an approved representative (eg, pharmacist), will ensure that all study drug is stored in a secured area, under recommended storage conditions and in accordance with applicable regulatory requirements. Zidovudine/lamivudine must be stored according to manufacturer's guidelines.

To ensure adequate records, all study drug will be accounted for in the case report form and drug accountability inventory forms as instructed by Pfizer. Unless otherwise authorized by Pfizer, all unallocated or unused drug supplies must be returned to Pfizer or its appointed agent (eg, CRO) at the end of the clinical trial. Subjects must return all bottles (including empty) to the Investigator, who will then return the bottles to Pfizer or an appointed agent (eg, CRO) for review of dosing records and destruction.

5.5. Concomitant Medication(s)

Zidovudine/lamivudine will be administered in open-label fashion and provided by the sponsor. Patients experiencing toxicity attributed to zidovudine and/or lamivudine would be able to substitute one or two alternative nucleoside analogues during the trial in consultation with the medical monitor. All substituted antiretroviral agents must be recorded on the CRF. No other anti-HIV therapy will be allowed while on study medication. Medications such as analgesics, anti-inflammatory agents, antibiotics and nutritional supplements may be used as needed for treatment of adverse events and preexisting conditions. Prophylaxis for *Pneumocystis carinii* pneumonia (PCP) is strongly recommended for subjects with CD4 cell counts ≤200 cells/mm^3 or who have had a prior episode of PCP. Routine immunizations are permitted, as well as the use of erythropoietin and/or G-CSF and/or blood transfusions in the appropriate clinical situation.

As UK-427,857 is a substrate for CYP3A4 inhibition, the following agents should not be co-administered during the study period due the risk of drug-drug interactions: ketoconazole, itraconazole, miconazole, clotrimazole, troleandomycin, nefazadone, clarithromycin, rifampin and rifabutin. Because of the restriction on rifampin use, patients who develop isolated pulmonary *M. tuberculosis* infection and require the use of rifampin will be kept in the study but off study drug (ie, efavirenz or UK-427,857).

The following agents should not be co-administered during the study period:

- Immunomodulators (for the treatment of HIV-1 infection; required agents such as interferon for the ongoing treatment of hepatitis C infection is permitted);
- Grapefruit or grapefruit juice;
- Over-the-counter medicines (except paracetamol and multivitamins) including St. John's Wort; or
- Other herbal or food supplements.

This list is not exhaustive and new therapies may arise that would need to be considered as exclusionary.

090000042732721.0\Approved\24-Aug-2004 11:39

0100000427327211.0\Approved\24-Aug-2004 11:39

5.6. Rescue Therapy

If a patient meets the criteria for treatment failure or discontinues for another reason (eg, pregnancy, adverse event) and requires an alternative regimen, they will be followed until the Week 96 visit as per protocol guidelines. The new regimen, selected by the Investigator based on the results of resistance testing at the time of failure, must be recorded in the CRF. For patients whose virus still remains CCR5-tropic and potentially sensitive to UK-427,857, study drug may be continued during this period. The Investigator, in consultation with the medical monitor, will evaluate the appropriateness of continued therapy with UK-427,857 based on ongoing review of clinical and laboratory parameters.

Women who become pregnant during the study period must be unblinded to study drug following end of study assessments. The Investigator, in consultation with the medical monitor, should decide on the optimal antiretroviral regimen on an individualized basis. Protocol-required procedures for follow-up must be performed unless contraindicated by pregnancy. Other appropriate pregnancy follow-up procedures related to perinatal care and neonatal outcome should be performed and noted in the CRF. A detailed fetal anomalies ultrasound scan should be performed between Weeks 12 and 16 of gestation and recorded in the CRF. In addition, the Investigator must complete a Pfizer pregnancy surveillance form. Infants will be enrolled into an in utero exposed infant follow-on study. This study will have regular assessments and require a development assessment at 18 months.

This protocol will be extended for a minimum of 3 years, following the last patient's last visit at Week 96. While the regulatory requirement for 2-year follow-up in this patient population will have been fulfilled, we intend to further evaluate the long-term safety and efficacy of UK-427,857, compared with efavirenz, in antiretroviral-naïve patients. During this time, patients will be monitored on an every 12-week schedule and the "On Study" procedures will be performed, apart from pharmacokinetic sampling.

6. TRIAL PROCEDURES

An overview of the schedule of study visits and procedures is presented as a flowchart in the Protocol Synopsis. Subjects will have study related visits with site personnel at the following timepoints:

- Screening: will occur within ~6 weeks prior to initial dosing with study medication.
- Randomization: will occur within 1 week of dosing with study medication.
- Baseline: Is the day that dosing with study medication will begin. Study procedures should be performed prior to the first dose.
- Weeks 2, 4 and every 4 weeks thereafter until Week 24.
- Every 8 weeks until Week 48.
- Every 12 weeks until Week 96.

Every attempt should be made to keep subjects to visit schedules ±4 days. It is anticipated that subjects may require additional unscheduled clinic visits. If a subject discontinues study therapy prior to the Week 96 visit, they will complete the End of Study procedures but will remain "in study off drug", keeping to the scheduled visits and procedures as outlined. The schedule of evaluations is listed below.

6.1. Screening Visit (Day –42 to Day –28)

- Written informed consent;
- Review of inclusion/exclusion criteria;
- Orthostatic blood pressure monitoring;
- Serum chemistry and hematology;
- CD4 and CD8 lymphocyte count determinations (absolute and percent);
- Hepatitis screening panel (hepatitis B core antibody and surface antigen/antibody, hepatitis C antibody);
- Plasma HIV-1 RNA level as determined by the Roche Amplicor HIV-1 MONITOR test, standard method;
- Plasma sample for HIV-1 co-receptor tropism phenotype as determined by the ViroLogic recombinant virus entry assay;
- Plasma sample for HIV-1 genotype and phenotype as determined by the ViroLogic PhenoSense™ GT assay; and
- Serum pregnancy testing for WOCBP (see Section 4.1).

6.2. Trial Period

6.2.1. Randomization Visit (Day -7 to Day -4)

Patients will have a randomization visit between Days -7 and -4 for all screening results to be reviewed and be randomized into the study if all the entry criteria are met. In addition, the following will be performed:

- Plasma HIV-1 RNA level as determined by the Roche Amplicor HIV-1 MONITOR test, standard method (If the results of the standard method are <400 copies/mL, the ultrasensitive method will automatically be performed); (Note: this result will not be used to determine eligibility).

6.2.2. Baseline (Day 1) Evaluation – *Prior to first dose*

- Medical history, including HIV-related diagnoses;
- Complete physical examination and vital signs (including orthostatic BP monitoring);
- Waist/Hip lipodystrophy measurements for morphology assessment (see Appendix C);

01000004273272\1.0\Approved\24-Aug-2004 11:39

0100000427327 2\1.0\Approved\24-Aug-2004 11:39

- Assessment of signs, symptoms and adverse events;
- Review of concomitant medications;
- Serum chemistry and hematology;
- Fasting Metabolic Assessment (total cholesterol, HDL/LDL, triglycerides, glucose, glycosylated hemoglobin);
- 12-lead electrocardiogram;
- Urinalysis;
- Hepatitis C virus RNA if hepatitis C virus antibody positive at screening;
- CD4 and CD8 lymphocyte count determinations (absolute and percent);
- Plasma HIV-1 RNA level as determined by the Roche Amplicor HIV-1 MONITOR test, standard method (If the results of the standard method are <400 copies/mL, the ultrasensitive method will automatically be performed);
- Plasma sample for HIV-1 co-receptor tropism phenotype as determined by the ViroLogic recombinant virus entry assay;
- Plasma sample stored for potential HIV-1 gp160 sequencing;
- Blood sample for preparation of two 1 mL plasma aliquots (frozen) and whole blood (17 mL) sample at ambient temperature for the purpose of PBMC and proviral DNA preparation and storage for future testing;
- Urine pregnancy testing for WOCBP (see Section 4.1). A positive urine test will require a confirmatory a serum pregnancy test;
- Nine mL blood sample for host genotyping;
- Thyroid Function Tests (free T4, TSH);
- CT scan of abdomen and thigh for subcutaneous and visceral fat determination on a subset of patients enrolled from selected centers;
- ACTG Symptom Distress Module to be completed by the patient; and
- Dispense Study medication.

6.2.3. Week 2 Visit

- Assessment of signs, symptoms and adverse events;
- Review of concomitant medications;
- Serum chemistry and hematology;
- Orthostatic BP monitoring (to be performed immediately prior to the first pharmacokinetic sample);
- Two 5 mL pharmacokinetic samples separated by at least 30 minutes;

- CD4 and CD8 lymphocyte count determinations (absolute and percent);
- Plasma HIV-1 RNA level as determined by the Roche Amplicor HIV-1 MONITOR test, standard method (If the results of the standard method are <400 copies/mL, the ultrasensitive method will automatically be performed);
- Plasma sample for HIV-1 co-receptor tropism phenotype as determined by the ViroLogic recombinant virus entry assay;
- Review dosing compliance; and
- Dispense Study medication (container from previous visit).

6.2.4. On Study Evaluations (Week 4, 8, 12, 16, 20, 32, 40, 60, 72 and 84)

- Targeted physical examination and vital signs;
- Assessment of signs, symptoms and adverse events;
- Review of concomitant medications;
- Serum chemistry and hematology;
- One 5 mL pharmacokinetic sample at all visits through Week 40;
- Hepatitis C virus RNA if hepatitis C virus antibody positive at screening (Week 12 only);
- CD4 and CD8 lymphocyte count determinations (absolute and percent);
- Plasma HIV-1 RNA level as determined by the Roche Amplicor HIV-1 MONITOR test, standard method (If the results of the standard method are <400 copies/mL, the ultrasensitive method will automatically be performed);
- Blood sample for preparation of two 1 mL plasma aliquots (frozen) stored for future testing;
- Urine pregnancy testing for WOCBP (see Section 4.1). A positive urine test will require a confirmatory a serum pregnancy test;
- Plasma sample for HIV-1 co-receptor tropism phenotype as determined by the ViroLogic recombinant virus entry assay at all visits for patients with HIV-1 RNA >500 copies/mL and upon treatment failure (as defined in the protocol) if this occurs;
- ACTG Symptom Distress Module to be completed by the patient, at Week 12 only;
- Plasma sample for HIV-1 genotype and phenotype as determined by the ViroLogic PhenoSense GT assay upon treatment failure only (as defined in the protocol);
- Plasma sample for potential HIV-1 gp160 sequencing upon treatment failure only (as defined in the protocol);
- Review dosing compliance; and
- Dispense Study medication.

0100000427327 2\1.0\Approved\24-Aug-2004 11:39

6.2.5. Week 24, 48 and 96 or Early Termination Visit

- Complete physical examination and vital signs;
- Assessment of signs, symptoms and adverse events;
- Waist/Hip lipodystrophy measurements for morphology assessment (see Appendix C);
- Review of concomitant medications;
- Serum chemistry and hematology;
- Fasting Metabolic Assessment (total cholesterol, HDL/LDL, triglycerides, glucose, glycosylated hemoglobin);
- 12-lead electrocardiogram and orthostatic BP monitoring (to be performed immediately prior to the first pharmacokinetic sample);
- Two 5 mL pharmacokinetic samples separated by at least 30 minutes (at Weeks 24 and 48 only);
- Urinalysis;
- Hepatitis C virus RNA if hepatitis C virus antibody positive at screening;
- CD4 and CD8 lymphocyte count determinations (absolute and percent);
- Plasma HIV-1 RNA level as determined by the Roche Amplicor HIV-1 MONITOR test, standard method (If the results of the standard method are <400 copies/mL, the ultrasensitive method will automatically be performed);
- Blood sample for preparation of two 1 mL plasma aliquots (frozen) and whole blood (17 mL) sample at ambient temperature for the purpose of PBMC and proviral DNA preparation and storage for future testing;
- Urine pregnancy testing for WOCBP (see Section 4.1). A positive urine test will require a confirmatory a serum pregnancy test;
- Plasma sample for HIV-1 co-receptor tropism phenotype as determined by the ViroLogic recombinant virus entry assay upon treatment failure (as defined in the protocol);
- Plasma sample for potential HIV-1 gp160 sequencing upon treatment failure (as defined in the protocol);
- Plasma sample for HIV-1 genotype and phenotype as determined by the ViroLogic PhenoSense GT assay upon treatment failure (as defined in the protocol);
- Thyroid Function Tests (free T4, TSH);
- CT scan of abdomen and thigh for subcutaneous and visceral fat determination on a subset of patients enrolled from selected centers (Week 96 only or at Early Termination on or after Week 48);ACTG Symptom Distress Module to be completed by the patient;
- Review dosing compliance; and

010000042732721.0\Approved\24-Aug-2004 11:39

- Dispense Study medication (exception of Week 96 visit).

6.3. Population Pharmacokinetics

During each of the Week 4, 8, 12, 16, 20, 24, 32 and 40 visits, one venous blood samples should be taken from each patient. During each of the Week 2 and 48 visits, two venous blood samples should be taken from each patient. The time of draw of the two samples should be as far apart as possible and should be separated by at least 30 minutes. It is recommended that the first sample be obtained as soon as the possible upon arrival, and the second immediately prior to leaving the clinic at each appointment.

Where possible, the samples at each visit should be obtained at different times post-dose relative to each other. To this end it is recommended that the:

- Week 2 and 8 visit appointments should be scheduled in the morning.
- Week 4, 16 and 40 visit appointments should be scheduled at around midday.
- Week 12 and 32 visit appointments should be scheduled in the afternoon.
- Week 20, 24 and 48 visits appointments should be scheduled in the morning.

0100000427327 2\1.0\Approved\24-Aug-2004 11:39

6.3.1. Population Pharmacokinetics: Dosing history

For the final pharmacokinetic analysis, it is important that the date and time of administration of the previous study drug dose before collection of the pharmacokinetic sample (PK dose), and the dose before that, be accurately recorded in the source documents and CRF at all visits. Similarly, it is important that the date and time that each blood sample is drawn is accurately recorded in the CRF.

6.3.2. Population Pharmacokinetics: Meal times history

The date and time of the last meal prior to the PK dose should also be recorded in the CRF.

6.4. Drug Assay

At a time determined by the study monitor, the samples will be transported in dry ice to Covance Central Laboratory and stored until being shipped to Tandem Analytics where they will be assayed for UK-427,857 using a previously validated method. Final UK-427,857 plasma concentration data will be transferred to the Pfizer Clinical Data System. All samples must be stored and shipped according to the procedures detailed in the Central Laboratory Manual.

6.5. Virology Testing

6.5.1. Viral load measurements

Viral load will be determined using the RT-PCR (Roche Amplicor v1.5) assay with a lower limit of detection of 400 copies/mL as standard. For samples with a reading of <400 copies/mL, the

ultrasensitive method with a lower limit of detection of 50 copies/mL will be used. Blood samples (10 mL) will be taken at the following timepoints:

- Screening;
- Randomization;
- Baseline;
- Weeks 2, 4, 8, 12, 16, 20, 24, 32, 40, 48, 60, 72, 84 and 96;
- Early Termination Visit.

Blood will be collected, processed, stored and shipped to Covance in accordance with the procedure documented in a separate sample handling document. Baseline will be calculated as the mean of the 3 pre-dose values, however, only the screening value will be used to determine eligibility for the study.

6.5.2. Virus tropism

Virus tropism will be determined using the ViroLogic PhenoSense™ Entry Assay. Samples will not be analyzed if the viral load is less than 500 copies/mL. Blood samples (10 mL) will be taken at the following time points:

- Screening;
- Baseline;
- Weeks 2, 4, 8, 12, 16, 20, 24, 32, 40, 48, 60, 72, 84 and 96;
- At the time of treatment failure;
- Early Termination Visit.

Blood will be collected, processed, stored and shipped in accordance with the procedure documented in a separate handling document. A part of the sample may be retained and archived for potential analysis of other virus tropism assays if deemed necessary.

V3 loop sequencing, alone or as part of gp160 sequencing, will be performed at baseline, Weeks 24, 48 and 96 and at treatment failure.

6.5.3. Viral resistance

Phenotypic and genotypic resistance to PIs, NRTIs and NNRTIs will be evaluated using the ViroLogic PhenoSense™ GT assay. Genotypic resistance to enfuvirtide will be determined using gp41 sequencing. Blood samples (10 mL) will be taken at the following timepoints:

- Screening;
- Weeks 24, 48 and 96;
- At the time of treatment failure;

0100000427327\1.0\Approved\24-Aug-2004 11:39

- Early Termination Visit.

Blood will be collected, processed, stored and shipped in accordance with the procedure documented in a separate handling document.

6.5.4. Virus susceptibility to UK-427,857

Blood samples (17 mL) will be taken to provide stored PBMCs and proviral DNA at the following timepoints:

- Baseline;
- Weeks 24, 48 and 96
- At the time of treatment failure;
- Early Termination Visit.

Blood will be collected, processed, stored and shipped in accordance with the procedure documented in a separate handling document after which PBMCs will be prepared and stored according to a standardized protocol. At time of treatment failure these samples will be used to attempt virus isolation and susceptibility testing according to a standardized protocol.

Other virus characteristics that could impact on susceptibility to UK-427,857 and virological response may also be evaluated on a subset of patient samples at baseline and at the time of treatment failure. These potentially will include gp160 sequencing, virus replication assays and virus subtyping.

6.5.5. Blood Pressure, Heart Rate and ECG Monitoring

Supine and standing BP and HR measurements will be recorded using a semi-automated sphygmomanometer, and a 12-lead ECG will be recorded at the following times:

- Screening (BP and HR only);
- Baseline;
- Weeks 2 (BP and HR only), 24, 48 and 96;
- Early Termination Visit.

Supine blood pressure will be recorded after 5 minutes lying down; patients will then sit for 2 minutes, followed by standing for 2 minutes after which a standing blood pressure will be recorded.

Patients with symptomatic postural hypotension at the screening or baseline visits will be excluded until such time as this has been appropriately managed. Patients with asymptomatic postural hypotension at the baseline visit will be monitored for 4 hours following the first dose of study drug and any adverse events recorded. Any adverse events felt potentially related to

01000004273272\1.0\Approved\24-Aug-2004 11:39

postural hypotension at this visit should be discussed immediately with the medial monitor or designee.

During the subsequent evaluation and treatment period, episodes of postural hypotension and potentially related events should be managed in the same fashion as other adverse events, with causality and severity assessed by the Investigator and a determination as to whether the event meets the criteria for a Serious Adverse Event and/or requires discontinuation of study drug. The Investigator and patient will assess the risk/benefit of continued dosing with study drug in consultation with the medical monitor.

6.5.6. Host Genotyping

In recognition of the fact that genetic variation in the CCR5 locus is well documented and that it is possible that the antiretroviral activity of UK-427,857 may vary between individuals based on their CCR5 genotype and in response to a request from regulators to include pharmacogenetic analysis in the development program. Prior to the first dose (of UK-427,857 or efavirenz) a 9 ml blood sample will be collected for non-anonymous genotyping. In this context, non-anonymous means that the genotyping results can be linked to the subject who donated the sample via the patient identifier. Initially, samples will be genotyped to determine the patient's CCR5 Δ32 status and for other CCR5 locus polymorphisms. A patient's CCR5 Δ32 genotyping results will be transferred to the appropriate investigator following completion of the trial if the patient requests the information. The results will only be forwarded on a per patient basis.

In addition, genotyping of drug metabolizing enzymes, drug transport proteins (for example CYP3A4, CYP3A5, MDR1) or other genes that might influence, safety, toleration or efficacy of UK-427,857 (for example, those involved in blood pressure homeostasis such as ADRA1A, ACE) may also be performed in the event of unusual patterns of response, or an unexplained excess of adverse events. If additional genotyping is performed these results will **ONLY** be used to assess the impact of genetic variation on response to UK-427,857 and will not be transferred back to the investigators. The blood samples will initially be stored at the central laboratory prior to being transferred to Pfizer Global Research and Development, 2800 Plymouth Road, Safety Sciences-Bldg 30 Rm 151C, Ann Arbor, MI 48105 USA or an appropriate contract laboratory for genotyping. The samples will be retained until all regulatory post approval commitments have been satisfied, after which they will be destroyed.

6.6. Subject Withdrawal

A subject may withdraw from the trial at any time at their own request, or they may be withdrawn at any time at the discretion of the investigator or sponsor for safety, behavioral, or administrative reasons. If a subject does not return for a scheduled visit, every effort should be made to contact the subject. In any circumstance, every effort should be made to document subject outcome, if possible.

If the subject withdraws consent, no further evaluations should be performed and no attempts should be made to collect additional data.

0100000427327 2\1.0\Approved\24-Aug-2004 11:39

The primary reason for a patient discontinuing from study drug or the clinical study will be recorded in the CRF. The investigator must determine the primary reason for discontinuation. Withdrawal due to adverse events must be distinguished from withdrawal due to insufficient efficacy. A discontinuation must be reported immediately to Pfizer medical monitor or his/her designated representative if it is due to a serious adverse event. If a subject is discontinued from the study or study treatment due to a drug-related adverse event, the subject must be followed until the adverse event has resolved or until the event is determined to be chronic or stable in nature. The End of Study procedures must be performed at the time of discontinuation (and prior to unblinding) from either study drug or from the clinical study.

All planned discontinuations must be discussed with the medical monitor or his/her designated representative prior to investigator unblinding. Pfizer personnel directly involved in the conduct of the study will remain blinded to these individual subjects who continue "in study off drug".

A subject may be withdrawn from study therapy at any time for any of the following reasons; however, they will remain " in study, off drug" and will be monitored as defined in the protocol specified guidelines until Week 96:

- An adverse event or laboratory abnormality requiring drug discontinuation;
- Pregnancy;
- Planned enrollment into another study (including, but not limited to intervention, laboratory, psychological, observational, or investigational drug, device, or biological studies);
- Treatment failure, defined as:
    - Failure to achieve HIV-1 RNA <400 copies/mL by Week 24;
    - An increase to at least 3 times the baseline (mean of all 3 values before start of dosing) plasma HIV-1 RNA level at the Week 2 visit or thereafter (confirmed by a second measurement taken no more than 14 days after the first measurement);
    - HIV-1 RNA $< 1.0 \log_{10}$ decrease from baseline (mean of all 3 values before start of dosing) at Week 4 or thereafter (confirmed by a second measurement taken no more than 14 days after the first measurement); or
    - An increase in HIV-1 RNA to detectable levels (≥1,000 copies/mL on two consecutive measurements) in subjects previously confirmed to have undetectable levels of <400 copies/mL (confirmed by a second measurement taken no more than 14 days after the first measurement).
- In the Investigator's opinion, it is in the subject's best interest;

In addition, subjects may be formally discontinued from the study for any of the following reasons:

- Subject's decision not to participate any further (withdrawal of consent);

01000004273272\1.0\Approved\24-Aug-2004 11:39

- In the Investigator's opinion, it is in the subject's best interest;
- The study is terminated by Pfizer;
- Lack of compliance with administration of study medication or with protocol procedures such that reliable safety and efficacy assessments are compromised.

7. ASSESSMENTS

7.1. Efficacy Assessments

All subjects will have antiviral activity assessed by HIV-1 PCR RNA levels and immunological status assessed by CD4 and CD8 counts at specified study visits. Blood samples for viral tropism and resistance testing will be drawn at specified study visits.

7.2. Safety Assessments

Safety will be assessed by spontaneous reports, physical examination and laboratory test results in all subjects who received at least one dose of study medication.

Safety assessments will be done at specified study visits and will include the following:

- Medical history
- Complete or symptom directed physical examination
- Vital signs
- Laboratory tests (eg, hepatitis testing, pregnancy testing, hematology, chemistry, urinalysis and fasting metabolic assessment).

Other assessments will include recording of concomitant medications and adverse events.

Abnormalities of any laboratory test considered to represent a significant danger to the patient will lead to immediate discontinuation of study drug. If a subject develops a Grade 3 or Grade 4 abnormality (with the exception of hypercholesterolemia, hypertriglyceridemia and asymptomatic CPK elevations), the Investigator will immediately discuss the case with the medical monitor. Study medication must be discontinued immediately unless the medical monitor and Investigator agree that there is an adequate explanation for the abnormality and the subject can be safely continued. These patients will be followed with appropriate medical management until there is a return to baseline values or a clinical diagnosis of an intercurrent illness has been established. Specific guidelines for the management of patients with abnormalities in hepatic enzymes are provided in Appendix B.

8. ADVERSE EVENT REPORTING

Following study drug discontinuation, adverse events will be considered as potentially related to study drug (UK-427,857 or efavirenz) until another medication is taken. Adverse events may then be ascribed as potentially related to this new medication.

01000004273272\1.0\Approved\24-Aug-2004 11:39

8.1. Adverse Events

All observed or volunteered adverse events regardless of treatment group or suspected causal relationship to the investigational product(s) will be recorded on the adverse event page(s) of the case report form (CRF).

For all adverse events, the investigator must pursue and obtain information adequate both to determine the outcome of the adverse event and to assess whether it meets the criteria for classification as a serious adverse event requiring immediate notification to Pfizer or its designated representative (see Section 8.4). For all adverse events, sufficient information should be obtained by the investigator to determine the causality of the adverse event. The investigator is required to assess causality and indicate that assessment on the CRF. Follow-up of the adverse event, after the date of therapy discontinuation, is required if the adverse event or its sequelae persist. Follow-up is required until the event or its sequelae resolve or stabilize at a level acceptable to the investigator and the Pfizer medical monitor or designee.

8.2. Definition of an Adverse Event

An adverse event is any untoward medical occurrence in a clinical investigation subject administered a product or medical device; the event need not necessarily have a causal relationship with the treatment or usage. Examples of adverse events include but are not limited to:

- Abnormal test findings;
- Clinically significant symptoms and signs;
- Changes in physical examination findings;
- Hypersensitivity;
- Progression/worsening of underlying disease;
- Lack of effect.

Additionally, they may include the signs or symptoms resulting from:

- Drug overdose;
- Drug withdrawal;
- Drug abuse;
- Drug misuse;
- Drug interactions;
- Drug dependency;
- Extravasation;
- Exposure *in utero*;

010000042732721.0\Approved\24-Aug-2004 11:39

- Lack of or insufficient clinical response, benefit, efficacy or therapeutic effect should not be recorded as an adverse event.

For sentinel HIV-related infections, as well as AIDS-defining opportunistic infections and malignancies, detailed information will be collected. A list of these is provided in Appendix A.

8.3. Abnormal Laboratory Findings

A designated central laboratory will perform laboratory safety tests. Any screening laboratory result outside the inclusion or exclusion criteria may be repeated. If repeat screening results remain outside the eligibility criteria, the subject should not be enrolled unless there is prior written agreement between the sponsor and the Investigator to allow an exemption from the inclusion or exclusion criteria. Laboratory safety tests will be performed at the following time points *(See Study Schedule/Flowchart)*

- Screening
- Randomization
- Day 1 Pre-dose
- Week 2
- On Study Evaluations (Week 4, 8, 12, 16, 20, 32, 40, 60, 72 and 84)
- Week 24, 48 and 96 or Early Termination Visit

8.3.1. Laboratory Tests will include:

1. **Hematology:** hemoglobin, RBC, hematocrit, WBC (including differentials) and platelet count.
2. **Clinical Chemistry:** total bilirubin (direct/indirect if elevated), total protein, albumin, uric acid, sodium, potassium, chloride, bicarbonate, BUN, creatinine, calcium, AST, ALT, lactate dehydrogenase, alkaline phosphatase, GGTP (if alkaline phosphatase elevated), creatine kinase and amylase and lipase (if amylase elevated). Fasting metabolic assessment, performed at baseline, Week 24, 48 and 96 or Early Termination, will include glucose (at all other times glucose will be non-fasting), glycosylated hemoglobin, total cholesterol, high-density lipoprotein cholesterol and triglycerides.
3. **Serum pregnancy test:** On women of childbearing potential (ie, females who have experienced menarche and who have not undergone successful surgical sterilization or is not post-menopausal). Serum pregnancy will be performed at the Screening visit and Urine test will be used at the following visits. A positive urine test will require a confirmatory serum pregnancy test.
4. **Hepatitis testing:** At screening will include hepatitis B surface antigen (HBsAg), hepatitis B surface antibody (HBsAb), hepatitis B core antibody (HBcAb) and hepatitis C antibody (anti-HCV). If HCV positive at screening, HCV RNA should be determined at baseline and Weeks 12, 24, 48 and 96 or Early Termination.

010000042732721.0\Approved\24-Aug-2004 11:39

5. **Blood:** Will be collected for thyroid function tests (free T4 and TSH), CD4/CD8 cell counts, plasma HIV-1 RNA, viral tropism and drug resistance assays (see Section 6.5) and pharmacokinetic testing (see Section 6.3).

   Blood will be collected, processed, stored and shipped in accordance with the procedures outlined in a separate handling protocol. A total of 8.5 mL of blood will be taken for clinical chemistry and 4 mL for hematology measurements.

6. **Urine:** Urinalysis will include color, specific gravity, pH, blood, protein, glucose, ketones, bilirubin and a microscopic examination, which will include casts, crystals, WBC and RBC.

7. **CSF:** Cerebrospinal fluid samples (CSF) for measurement of UK-427,857 levels will be collected in patients who undergo diagnostic or therapeutic lumbar- or ventricular punctures during the study (see Appendix D).

8.3.2. Follow Up of Laboratory Test Abnormalities:

Abnormalities of any laboratory test considered to represent a significant danger to the patient will lead to immediate discontinuation of study drug. If a subject develops a Grade 3 or Grade 4 abnormality (with the exception of hypercholesterolemia, hypertriglyceridemia, and asymptomatic CPK elevations), the Investigator will immediately discuss the case with the medical monitor. Study medication must be discontinued immediately unless the medical monitor and Investigator agree that there is an adequate explanation for the abnormality and the subject can be safely continued. These patients will be followed with appropriate medical management until there is a return to baseline values or a clinical diagnosis of an intercurrent illness has been established. Specific guidelines for the management of patients with abnormalities in hepatic enzymes are provided in Appendix B.

The results of all laboratory tests required by the protocol will be recorded in the subject's case report form. All clinically important abnormal laboratory tests occurring during the study will be repeated at appropriate intervals until they return either to baseline or to a level deemed acceptable by the investigator and the Pfizer clinical monitor (or his/her designated representative), or until a diagnosis that explains them is made.

The criteria for determining whether an abnormal objective test finding should be reported as an adverse event are as follows:

- Test result is associated with accompanying symptoms, and/or
- Test result requires additional diagnostic testing or medical/surgical intervention, and/or
- Test result leads to a change in trial dosing or discontinuation from the study, significant additional concomitant drug treatment, or other therapy, and/or
- Test result is considered to be an adverse event by the investigator or sponsor.

Merely repeating an abnormal test, in the absence of any of the above conditions, does not constitute an adverse event. Any abnormal test result that is determined to be an error does not require reporting as an adverse event.

010000042732721\1.0\Approved\24-Aug-2004 11:39

0100000427327\1.0\Approved\24-Aug-2004 11:39

8.4. Serious Adverse Events

A serious adverse event or serious adverse drug reaction is any untoward medical occurrence at any dose that:

- Results in death;
- Is life-threatening;
- Requires inpatient hospitalization or prolongation of existing hospitalization;
- Results in persistent or significant disability/incapacity;
- Results in congenital anomaly/birth defect.

Medical and scientific judgment should be exercised in determining whether an event is an important medical event. An important medical event may not be immediately life-threatening and/or result in death or hospitalization. However, if it is determined that the event may jeopardize the subject and may require intervention to prevent one of the other outcomes listed in the definition above, the important medical event should be reported as serious.

Examples of such events are intensive treatment in an emergency room or at home for allergic bronchospasm; blood dyscrasias or convulsions that do not result in hospitalization; or development of drug dependency or drug abuse.[*]

8.5. Hospitalization

Adverse events reported from clinical trials associated with hospitalization or prolongation of hospitalization are considered serious. Any initial admission (even if less than 24 hours) to a healthcare facility meets these criteria. Admission also includes transfer within the hospital to an acute/intensive care unit (eg, from the psychiatric wing to a medical floor, medical floor to a coronary care unit, neurological floor to a tuberculosis unit).

Hospitalization does not include the following:

- Rehabilitation facilities;
- Hospice facilities;
- Respite care (eg, caregiver relief);
- Skilled nursing facilities;
- Nursing homes;
- Routine emergency room admissions;
- Same day surgeries (as outpatient/same day/ambulatory procedures).

[*] 21CFR 312.32

Hospitalization or prolongation of hospitalization in the absence of a precipitating, clinical adverse event is not in itself a Serious Adverse Event. Examples include:

- Admission for treatment of a preexisting condition not associated with the development of a new adverse event or with a worsening of the preexisting condition (eg, for work-up of persistent pre-treatment lab abnormality);
- Social admission (eg, subject has no place to sleep);
- Administrative admission (eg, for yearly physical exam);
- Protocol-specified admission during a clinical trial (eg, for a procedure required by the trial protocol);
- Optional admission not associated with a precipitating clinical adverse event (eg, for elective cosmetic surgery);
- Pre-planned treatments or surgical procedures should be noted in the baseline documentation for the entire protocol and/or for the individual subject.

Diagnostic and therapeutic non-invasive and invasive procedures, such as surgery, should not be reported as adverse events. However, the medical condition for which the procedure was performed should be reported if it meets the definition of an adverse event. For example, an acute appendicitis that begins during the adverse event reporting period should be reported as the adverse event, and the resulting appendectomy should be recorded as treatment of the adverse event.

010000042732721.0\Approved\24-Aug-2004 11:39

8.6. Severity Assessment

If required on the adverse event case report forms, the investigator will use the adjectives MILD, MODERATE, SEVERE or VERY SEVERE to describe the maximum intensity of the adverse event. For purposes of consistency, these intensity grades are defined as follows:	
MILD	Events which are usually transient, requiring no special treatment, and do not interfere with the patient’s daily activities.
MODERATE	Events which introduce a low level of inconvenience or concern to the patient and may interfere with daily activities, but are usually ameliorated by simple therapeutic measures.
SEVERE	Events that interrupt the patient’s usual daily activity and traditionally require systemic drug therapy or other treatment.
VERY SEVERE	Events which are unacceptable and intolerable or which are irreversible or cause the patient to be in imminent danger of death.

Note the distinction between the severity and the seriousness of an adverse event. A severe event is not necessarily a serious event. For example, a headache may be severe (interferes significantly with subject's usual function) but would not be classified as serious unless it met

one of the criteria for serious adverse events, listed above. See Appendix E for a listing of grading severities of adverse events.

8.7. Exposure In Utero

For investigational products within clinical trials and for marketed products, an *exposure in-utero* (EIU) occurs if a female becomes, or is found to be, pregnant either while receiving or having been exposed to (eg, environmental) an investigational medication or product, or the female becomes, or is found to be, pregnant after discontinuing and/or being exposed to the investigational medication or product.

If any trial subject becomes or is found to be pregnant while receiving an investigational medication/product, the investigator must submit this information to Pfizer medical monitor or designee on an Exposure in Utero Form. This must be done irrespective of whether an adverse event has occurred and within 24 hours of awareness of the pregnancy. The information submitted should include the anticipated date of delivery (see below for information related to induced termination of pregnancy).

The investigator will follow the subject until completion of the pregnancy or until pregnancy termination (ie, induced abortion) and then notify the Pfizer medical monitor or designee of the outcome. The investigator will provide this information as a follow up to the initial Exposure in Utero Form. The reason(s) for an induced abortion should be specified. An EIU report is not created when an ectopic pregnancy report is received since this pregnancy is not usually viable. Rather, an SAE case is created with the event of ectopic pregnancy.

If the outcome of the pregnancy meets the criteria for immediate classification as a serious adverse event (ie, spontaneous abortion, stillbirth, neonatal death, or congenital anomaly [including that in an aborted fetus, stillbirth or neonatal death]), the investigator should follow the procedures for reporting serious adverse events.

In the case of a live birth, the "normality" of the newborn can be assessed at the time of birth (ie, no minimum follow-up period of a presumably normal infant is required before an Exposure in Utero Form can be completed). The "normality" of an aborted fetus can be assessed by gross visual inspection, unless pre-abortion test findings are suggestive of a congenital anomaly.

Additional information about pregnancy outcomes that are classified as serious adverse events follows:

- "Spontaneous abortion" includes miscarriage and missed abortion.
- All neonatal deaths that occur within 1 month of birth should be reported, without regard to causality, as serious adverse events. In addition, any infant death after 1 month that the investigator assesses as possibly related to the in utero exposure to the investigational medication should be reported.

010000042732721.0\Approved\24-Aug-2004 11:39

8.8. Discontinuations (See also Subject Withdrawal, Section 6.6)

The reason for a subject discontinuing from the trial will be recorded in the CRF. A discontinuation occurs when an enrolled subject ceases participation in the study, regardless of the circumstances, prior to completion of the protocol. The investigator must determine the primary reason for discontinuation. Withdrawal due to adverse event should be distinguished from withdrawal due to insufficient response, according to the definition of adverse event noted earlier, and recorded on the appropriate adverse event CRF page.

When a discontinuation is due to a serious adverse event, the serious adverse event must be reported in accordance with the reporting requirements defined below.

8.9. Eliciting Adverse Event Information

The investigator is to report all directly observed adverse events and all adverse events spontaneously reported by the trial subject. In addition, each trial subject will be questioned about adverse events at each clinic visit. The question asked will be "Since your last clinic visit have you had any health problems?"

8.10. Reporting Requirements (Serious and Nonserious)

0100000427327\1.0\Approved\24-Aug-2004 11:39

Each adverse event is to be classified by the investigator as serious or nonserious. This classification determines the reporting procedures to be followed. If a serious adverse event occurs, expedited reporting will follow local and international regulations, as appropriate. SAEs are reportable from the time that the subject provides informed consent, which is obtained prior to the subject's participation in the clinical trial, ie, prior to undergoing any trial-related procedure and/or receiving investigational product, through and including 28 calendar days after the last administration of the investigational product. Any serious adverse event occurring at any other time after completion of the study must be promptly reported if a causal relationship to study drug is suspected.

If a serious adverse event occurs, the Pfizer medical monitor or designee is to be notified within 24 hours of awareness of the event by the investigator. In particular, if the serious adverse event is fatal or life-threatening, notification to the Pfizer medical monitor or designee must be made immediately, irrespective of the extent of available adverse event information. This timeframe also applies to additional new information (follow-up) on previously forwarded serious adverse event reports.

In the rare event that the investigator does not become aware of the occurrence of a serious adverse event immediately (eg, if an outpatient trial subject initially seeks treatment elsewhere), the investigator is to report the event within 24 hours after learning of it and document the time of his/her first awareness of the adverse event.

For all serious adverse events, the investigator is obligated to pursue and provide information to the Pfizer medical monitor or designee in accordance with the timeframes for reporting specified above. In addition, an investigator may be requested by the Pfizer medical monitor or designee

to obtain specific additional follow-up information in an expedited fashion. This information may be more detailed than that captured on the adverse event case report form. In general, this will include a description of the adverse event in sufficient detail to allow for a complete medical assessment of the case and independent determination of possible causality. Information on other possible causes of the event, such as concomitant medications and illnesses must be provided. In the case of a subject death, a summary of available autopsy findings must be submitted as soon as possible to Pfizer or its designated representative.

The investigator's assessment of causality must be provided. An investigator's causality assessment is the determination of whether there exists a reasonable possibility that the investigational product caused or contributed to an adverse event. If the investigator's final determination of causality is unknown and the investigator does not know whether or not study drug caused the event, then the event will be handled as "related to study drug" for reporting purposes. If the investigator's causality assessment is "unknown but not related to study drug", this should be clearly documented on study records. In addition, if the investigator determines the adverse event is associated with trial procedures, the investigator must record this causal relationship in the source documents and CRF, as appropriate, and report such an assessment in accordance with the serious adverse event reporting requirements, if applicable.

All adverse events will be reported on the adverse event page(s) of the CRF. It should be noted that the form for collection of serious adverse event information is not the same as the adverse event CRF. Where the same data are collected, the forms must be completed in a consistent manner. For example, the same adverse event term should be used on both forms. Adverse events should be reported using concise medical terminology on the CRFs as well as on the form for collection of serious adverse event information.

Nonserious adverse events are to be reported on the adverse event CRFs, which are to be submitted to Pfizer as specified in the adverse event report submission procedure for this protocol.

01000004273272\1.0\Approved\24-Aug-2004 11:39

8.11. Additional Safety Information

No mechanistically specific antagonists for UK-427,857 or efavirenz are available and standard supportive measures should be used in the case of excessive pharmacological effects.

9. DATA ANALYSIS/STATISTICAL METHODS

Detailed methodology for summary and statistical analyses of the data collected in this trial will be documented in an Analysis Plan, which will be dated and maintained by the sponsor. This document may modify the plans outlined in the protocol; however, any major modifications of the primary endpoint definition and/or its analysis will also be reflected in a protocol amendment.

9.1. Sample Size Determination

A total of 1071 subjects will be randomized in a 1:1:1 ratio (357 on UK-427,857 300 mg QD, UK-427,857 300 mg BID and efavirenz 600 mg QD). Under the assumption that the response

rate will be 75% for percentage of subjects with HIV-1 RNA levels <50 copies/mL in each group, with a 1-sided significance level of 0.0125 (Bonferroni adjustment for multiple comparisons) and a non-inferiority margin of -10% (ie, the lower bound of the 1-sided confidence interval will be above -10%) there will be 80% power to demonstrate non-inferiority. For the analysis of percentage of subjects with HIV-1 RNA levels <400 copies/mL in each group, with the same significance level and non-inferiority margin as above, there will be 86% power to demonstrate non-inferiority. If a UK-427,857 dosing arm is dropped following DSMB review at the interim analysis, the Bonferroni adjustment will still be made at the formal endpoint for the remaining dose. A response is defined as undetectable plasma HIV RNA levels using the Roche Amplicor HIV–1 MONITOR Test, Standard assay ie, viral load <400 copies/mL. This is the primary efficacy endpoint.

9.2. Efficacy Analysis

9.2.1. Analysis Populations

All tables and figures in this report will be based on one of three populations. Efficacy tables will be based on the “Full Analysis Set” and “Per protocol set”. Safety tables will be based on the "Safety Population”.

010000042732721\1.0\Approved\24-Aug-2004 11:39

The Full Analysis Set will consist of all randomized patients who receive at least one dose of study medication. It will use the intention to treat (ITT) approach.

The per protocol set will consist of all randomized patients who meet the following criteria:

- Receive at least one dose of study medication,
- Treated for at least 14 days or discontinued before this time due to treatment failure,
- More than 80% compliant with randomized treatment,
- No violation of any inclusion or exclusion criteria, which would affect efficacy (such as tropism status).

It will use the intention to treat (ITT) approach.

The Safety Population will consist of all randomized patients who received at least one dose of study medication. Patients will be included in the dose group they actually receive.

9.2.2. Analysis of Primary Endpoint

9.2.2.1. Primary Endpoint

Percentages of patients with viral load undetectable by the standard and more sensitive methods (<400 copies/mL and <50 copies/mL) are the primary efficacy endpoint.

A step down procedure will be used with percentage <400 tested first, and if non-inferiority is demonstrated then percentage <50 will be tested.

The primary efficacy time point is 48 weeks. This analysis will occur after all randomized subjects have reached the end of 48 weeks or discontinued prior to reaching 48 weeks. The primary efficacy variable will also be analyzed at 24 weeks and 96 weeks.

9.2.2.2. Method of Analysis

The percentage of subjects with HIV-1 RNA levels <400/<50 copies/mL at Week 48 will be summarized for the ITT population at each visit and using a last observation carried forward (LOCF) approach. One-sided 98.75% confidence intervals for the difference in percentages between each of the UK-427,857 dose groups and the efavirenz control arm at 48 weeks will be provided using the normal approximation. These will be adjusted based on the randomization strata.

Only those data collected during the blinded treatment phase will be used for the primary analysis

9.2.3. Analysis of Secondary Endpoints

9.2.3.1. Secondary Endpoints

Plasma HIV-1 RNA levels will be determined by the Roche Amplicor HIV-1 MONITOR Test, Standard assay. If the results of the standard method are <400 copies/mL the ultrasensitive assay will automatically be performed. For the analysis, HIV-1 RNA level for each subject at each time point will be defined based on results from the standard assay when the value is ≥400 copies/mL, and based on the results from the ultrasensitive assay when standard assay result is <400 copies/mL. Any HIV-1 RNA value <50 copies/mL (the LOQ from ultrasensitive assay) will be treated as 49.

010000042732721.0\Approved\24-Aug-2004 11:39

The following secondary efficacy variables will be analyzed:

- Percentage of subjects with HIV-1 RNA levels <400 copies/mL analyzed using logistic regression
- Percentage of subjects with HIV-1 RNA levels <50 copies/mL analyzed using logistic regression
- Change from baseline in $\log_{10}$ transformed HIV-1 RNA levels
- Time-Averaged Difference (TAD) in $\log_{10}$ transformed HIV-1 RNA levels
- Change from baseline in CD4 cell count
- Change from baseline in CD8 cell count
- Time to virological failure
- Association between baseline resistance and virological response
- Changes in genotype, phenotype and/or tropism in treatment failures after Week 4

All secondary variables will be analyzed at Weeks 48 and 96. All will also be analyzed at Week 24, apart from time to virological failure. Data summaries of the primary and most important secondary variables will be reviewed by the DSMB at this time point. No individual patient data will be communicated to Pfizer personnel.

9.2.3.2. Method of Analysis

Percentage of subjects with HIV-1 RNA levels <400/<50 copies/mL will be analyzed using logistic regression including baseline viral load level (<100,000 versus ≥100,000 copies/mL) (randomization strata) and geographic region (Northern Hemisphere/Southern Hemisphere) as factors.

Change from baseline to in $\log_{10}$ transformed HIV-1 RNA levels and Time-Averaged Difference (TAD) in $\log_{10}$ transformed HIV-1 RNA levels will be analyzed using an ANCOVA model with baseline viral load level (randomization strata), geographic region and treatment arm as the main effects. The least squares mean treatment difference between each UK-427,857 dose group and efavirenz will be presented.

This analysis will include only those subjects with an assessment of viral load at baseline and while on treatment. A sensitivity analyses will be performed by imputing no change from baseline for subjects with missing baseline or no viral load assessment on treatment.

Baseline will be calculated as the mean of all 3 values before start of dosing. These are collected at screening, randomization and immediately pre-dose. If 1 or 2 values are missing baseline is calculated from those present. Baseline is only missing if all 3 values are missing. All values are $\log_{10}$ transformed before the calculation.

Change from Baseline in CD4 and CD8 cell count will be analyzed using an ANCOVA model with baseline CD4 or CD8 cell count respectively, geographic region and treatment arm as the main effects.

If the assumption of normality is seriously violated in any of the ANCOVA analyses then the treatment effect will be assessed using non-parametric methods and non-parametric confidence intervals for the difference between treatments will be presented.

Time to virological failure (loss-of-virological-response) will be based on following algorithm:

Day 1 for this analysis is defined as the first occasion on which a subject had confirmed HIV-1 RNA levels below 400 copies/mL, ie, on the first of 2 consecutive visits. Time to virological failure is time from Day 1 until virological failure as defined below.

1. For 2. and 3. below discard all visits with no data. In what follows, a visit means a visit with an observed viral load. Viral load data from all available visits, including off-schedule visits and post Week 48 visits, should be included for the calculation.

01000004273272\1.0\Approved\24-Aug-2004 11:39

2. If a subject had never achieved a confirmed HIV-1 RNA level below 400 copies/mL (on 2 consecutive visits) before the following events, then this subject will be considered to have failed at Time 0:
    a. Death
    b. Permanent discontinuation of the test drug or loss to follow-up
    c. Introduction of a new anti-retroviral drug to the regimen, with the exception of changing a drug in the background therapy to a drug of the same class due to either toxicity or intolerance that is attributed to the background drugs, but not the test drug or its control
    d. Last available visit
    e. Entered open label due to early non-response or rebound (treatment failure criteria as described in protocol)
3. For all subjects who had confirmed HIV-1 RNA levels below 400 copies/mL, ie, on 2 consecutive visits, the time of failure is the earliest time when a specific event had occurred. Those events are modifications in 4) and are listed below:
    a. Death
    b. Permanent discontinuation of the test drug or loss to follow-up
    c. The event as described in 2c
    d. Entered open label due to early non-response or rebound (treatment failure criteria as described in section 3 of the protocol)
    e. Confirmed HIV-1 RNA levels above or equal to 400 copies/mL, defined as HIV-1 RNA levels from 2 consecutive visits are greater than or equal to 400 copies/mL or one visit greater than or equal to 400 copies/mL followed by permanent discontinuation of the test drug or loss to follow-up
4. If the time of virological failure defined above is immediately preceded by a single missing scheduled visit or multiple consecutive missing scheduled visits, then the time of virological failure is replaced by the first time of such missing visits.

A subject who has not failed during the study will be censored at the last available visit.

Time to virological failure will be summarized using Kaplan-Meier curves and the difference between the UK-427,857 arms and the control arm will be analyzed using a log-rank test, stratified by randomization strata.

Time of virological response is the time from Day 1 to the first of the 2 consecutive visits with HIV-1 RNA levels <400 copies/mL. Patients who never respond during the treatment will be censored at the last available observation.

The resistance profile at time of treatment failure will be summarized. Baseline V3 loop genotype and tropism phenotype will be summarized.

0100000427327 2\1.0\Approved\24-Aug-2004 11:39

9.3. Analysis of Other Endpoints

9.3.1. Pharmacokinetic Sampling and Population PK

A population analysis of time versus plasma concentration data of UK-427,857 will be performed using the nonlinear mixed effects modeling approach. The software NONMEM (UCSF, California, USA) will be used to derive the population mean (and variance) values for specific pharmacokinetic parameters. Additionally, a relationship between pharmacokinetic parameters (or dose) and efficacy, as well as adverse events, will be investigated.

In the pharmacokinetic (and pharmacodynamic) analysis a number of covariates will be tested and incorporated into the structural model if shown to significantly improve the model's ability to describe the data. The final PK/PD model for UK-427,857 will be obtained from this "full" model using only the covariate relationships that are thought to result in clinically significant alterations in drug pharmacokinetics and/ or pharmacodynamics.

A more detailed description of the methodology to be followed is given in the "Population Pharmacokinetic/Pharmacodynamic Analysis Plan for UK-427,857 Phase 2b/Phase 3 Data".

9.4. Safety Analysis

Safety will be assessed by spontaneous reports, physical examination, and laboratory test results in all subjects who received at least one dose of study medication.

9.5. Interim Analysis

Every 12-16 weeks a DSMB will meet to discuss the results from this and the 3 other studies in the UK-427,857 program. Summaries including demography, adverse events, discontinuations due to adverse events, viral load, CD4 cell count and tropism status will be provided by an independent Statistical Data Analysis Center (SDAC) statistician to the DSMB.

Additionally for this study the DSMB will review the results following treatment of 75 to 100 patients for at least 8 weeks. No formal statistical tests will be performed. If the DSMB feels that either of the doses of UK-427,857 show substantial evidence of harm to patients, then the DSMB will recommend ending recruitment to this treatment arm.

A formal interim analysis will be performed at the end of the run-in phase, when 204 patients (approximately 68 per treatment group) have been treated for 16 weeks. Non-inferiority of each UK-427,857 regimen in Time-Averaged Difference (TAD) in viral load, as well as response rate (percentage with HIV-1 RNA less than 400 copies/mL), versus the efavirenz regimen will be assessed. An analysis of covariance model will be fitted including treatment, baseline viral load stratum and geographic region. If either of the doses of UK-427,857 fails to demonstrate non-inferiority, and the DSMB feels there is enough evidence to justify dropping this dose from the study, then the DSMB will recommend ending recruitment to this treatment arm. The study will not be discontinued to make a claim for efficacy.

01000004273272\1.0\Approved\24-Aug-2004 11:39

With 68 patients per treatment group, assuming a standard deviation of 0.8 and with a 1-sided significance level of 0.025 (no adjustment for multiple comparisons at this interim analysis) and a non-inferiority margin of -0.5 $\log_{10}$ copies/mL (ie, the lower bound of the 1-sided confidence interval will be above –0.5) there will be 95% power to demonstrate non-inferiority between each UK-427,857 arm and efavirenz. Assuming a 90% response rate for percentage of subjects with HIV-1 RNA levels <400 copies/mL there is over 95% power to show non-inferiority with a non-inferiority margin of -20%.

Recruitment will continue during the formal interim analysis review period provided that the overall blinded treatment failure (ie, subjects who meet one of the prespecified stopping rules) rate does not exceed 7% (as this could potentially be observed with a >20% failure rate in a single arm) and/or if the DSMB review of 75 to 100 patients at 8 weeks confirms that continuation of the trial is appropriate. In the event of treatment failure rate exceeding 7% at any time prior to the DSMB 8 week review, recruitment will be suspended until the DSMB has completed their review.

At all other DSMB meetings there will be no stopping rule based on virological response, although the DSMB may recommend terminating the study if they feel that continuing would compromise patient safety. Patients will be protected by individual patient stopping rules based on treatment failure. All Pfizer personnel responsible for the clinical trial conduct will remain blinded to the data provided to the DSMB.

010000042732721.0\Approved\24-Aug-2004 11:39

9.6. Data Monitoring Committee

To help assure the safety of study participants, an independent Data Safety Monitoring Board (DSMB) will be formed. The DSMB will be responsible for evaluating the progress of the trial including periodic assessments of efficacy and safety data. In addition, the DSMB will make recommendations concerning continuation, termination or other modifications of the trial based on the observed beneficial or adverse effects of the treatment under study.

The DSMB will be made up of seven members with a Chairperson and statistician. A quorum will consist of 5 members. There will be both an open and closed portion of all DSMB meetings. The DSMB will report its recommendation to the Sponsor Management Committee. In reports prepared for the drug safety monitoring board, treatments will be labeled “A”, “B” and “C”. The assignment of actual assigned treatment to these labels will not be contained in the report; however, the convention will be consistent between reports. If the DSMB chooses to have the actual treatment assignments disclosed, a vote could be taken to authorize such disclosure. A full description of the DSMB process is detailed in the UK-427, 857 DSMB Charter.

The DSMB will review the results following treatment of 75 to 100 patients for at least 8 weeks. No formal statistical tests will be performed. If the DSMB feels that either of the doses of UK-427,857 show substantial evidence of harm to patients, then the DSMB will recommend ending recruitment to this treatment arm.

A formal interim analysis will be performed at the end of the run-in phase, when 204 patients have been treated for 16 weeks. Non-inferiority of each UK-427,857 regimen in Time-Averaged Difference (TAD) in viral load versus the efavirenz regimen will be assessed. If either of the doses of UK-427,857 fails to demonstrate non-inferiority, and the DSMB feels there is enough evidence to justify dropping this dose from the study, then the DSMB will recommend ending recruitment to this treatment arm. The study will not be discontinued to make a claim for efficacy.

10. QUALITY CONTROL AND QUALITY ASSURANCE

During trial conduct, Pfizer or its agent will conduct periodic monitoring visits to ensure that the protocol and GCPs are being followed. The monitors may review source documents to confirm that the data recorded on CRFs is accurate. The investigator and institution will allow Pfizer monitors or its agents and appropriate regulatory authorities direct access to source documents to perform this verification.

The trial site may be subject to review by the institutional review board (IRB)/independent ethics committee (IEC), and/or to quality assurance audits performed by Pfizer, and/or to inspection by appropriate regulatory authorities from the US or other countries.

It is important that the investigator(s) and their relevant personnel are available during the monitoring visits and possible audits or inspections and that sufficient time is devoted to the process.

11. DATA HANDLING AND RECORD KEEPING

11.1. Case Report Forms / Electronic Data Record

As used in this protocol, the term case report form (CRF) should be understood to refer to either a paper form or an electronic data record or both, depending on the data collection method used in this trial.

A CRF is required and should be completed for each included subject. The completed original CRFs are the sole property of Pfizer and should not be made available in any form to third parties, except for authorized representatives of Pfizer or appropriate regulatory authorities, without written permission from Pfizer.

It is the investigator's responsibility to ensure completion and to review and approve all CRFs. CRFs must be signed by the investigator or by an authorized staff member. These signatures serve to attest that the information contained on the CRFs is true. At all times, the investigator has final personal responsibility for the accuracy and authenticity of all clinical and laboratory data entered on the CRFs. Subject source documents are the physician's subject records maintained at the trial site. In most cases, the source documents will be the hospital's or the physician's chart. In cases where the source documents are the hospital or the physician's chart, the information collected on the CRFs must match those charts.

0100000427327\1.0\Approved\24-Aug-2004 11:39

In some cases, the CRF may also serve as the source document. In these cases, Pfizer and the investigator must prospectively document which items will be recorded in the source documents and for which items the CRF will stand as the source document.

11.2. Record Retention

To enable evaluations and/or audits from regulatory authorities or Pfizer, the investigator agrees to keep records, including the identity of all participating subjects (sufficient information to link records, eg, CRFs and hospital records), all original signed informed consent forms, copies of all CRFs, source documents, and detailed records of treatment disposition. The records should be retained by the investigator according to ICH, local regulations, or as specified in the Clinical Study Agreement, whichever is longer.

If the investigator relocates, retires, or for any reason withdraws from the trial, Pfizer should be prospectively notified. The trial records must be transferred to an acceptable designee, such as another investigator, another institution, or to Pfizer. The investigator must obtain Pfizer's written permission before disposing of any records, even if retention requirements have been met.

12. ETHICS

010000042732721.0\Approved\24-Aug-2004 11:39

12.1. Institutional Review Board (IRB)/Independent Ethics Committee (IEC)

It is the responsibility of the investigator to obtain prospective approval of the trial protocol, protocol amendments, Clinical Pharmacogenomics Supplement (if applicable), informed consent forms, and other relevant documents, eg, advertisements, if applicable, from the IRB/IEC. All correspondence with the IRB/IEC should be retained in the Investigator File. Copies of IRB/IEC approvals should be forwarded to Pfizer.

The only circumstance in which an amendment may be initiated prior to IRB/IEC approval is where the change is necessary to eliminate apparent immediate hazards to the subjects. In that event, the investigator must notify the IRB/IEC and Pfizer in writing within 5 working days after the implementation.

12.2. Ethical Conduct of the Trial

The trial will be performed in accordance with the protocol, International Conference on Harmonization Good Clinical Practice guidelines, and applicable local regulatory requirements and laws.

12.3. Subject Information and Consent

The informed consent form must be agreed to by Pfizer and the IRB/IEC and must be in compliance with ICH GCP, local regulatory requirements, and legal requirements.

The investigator must ensure that each trial subject, or his/her legally acceptable representative, is fully informed about the nature and objectives of the trial and possible risks associated with participation. The investigator will obtain written informed consent from each subject or the subject's legally acceptable representative before any study-specific activity is performed. The informed consent form used in this trial, and any changes made during the course of the trial, must be prospectively approved by both the IRB/IEC and Pfizer before use. The investigator will retain a copy of each subject's signed consent form.

13. SPONSOR DISCONTINUATION CRITERIA

Premature termination of this clinical trial may occur because of a regulatory authority decision, change in opinion of the IRB/IEC, drug safety problems, or at the discretion of Pfizer. In addition, Pfizer retains the right to discontinue development of UK-427,857 at any time.

Pfizer reserves the right to discontinue the trial prior to inclusion of the intended number of subjects, but intends only to exercise this right for valid scientific or administrative reasons. After such a decision, the investigator must contact all participating subjects within 24 hours. As directed by Pfizer, all trial materials must be collected and all CRFs completed to the greatest extent possible.

14. PUBLICATION OF STUDY RESULTS

Publication of study results is discussed in the Clinical Study Agreement.

01000004273272\1.0\Approved\24-Aug-2004 11:39

15. REFERENCES

1 Huang Y, Paxton WA, Wolinsky SM, Neumann AU, et al. The role of a mutant CCR5 allele in HIV-1 transmission and disease progression. Nat Med. 1996; 2(11):1240-3.

2 Meyer L, Magierowska M, Hubert JB, Rouzioux C, et al. Early protective effect of CCR-5 Δ-32 heterozygosity on HIV-1 disease progression: Relationship with viral load. AIDS 1997;11: f73-8.

3 D'Souza MP, Harden VA. Chemokines and HIV-1 second receptors. Nat Med 1996; 2:1293-1300.

4 UNAIDS Global Epidemic Report, December 2003.

5 Pozniak AI, Fatkenheuer G, Johnson M, et al. Effect of short-term monotherapy with UK-427,857 on viral load in HIV-infected patients. 43rd Annual Interscience Conference on Antimicrobial Agents and Chemotherapy, Sep 2003, Chicago, IL: Abstract 443

6 Reynes J, Rouzier R, Kanouni T, et al. SCH-C: safety and antiviral effects of a CCR5 receptor antagonist in HIV-1 infected subjects. 9th Conference on Retroviruses and Opportunistic Infections, Feb 2002, Seattle, WA: Abstract 1.

7 Schurmann D, Rouzier R, Nougarede R, et al. SCH-D: antiviral activity of a CCR5 receptor antagonist. 11th Conference on Retroviruses and Opportunistic Infections, Feb 2004, San Francisco, CA: Abstract 140

8 Pfizer, data on file.

9 Pfizer, data on file

10 Pfizer, data on file.

11 Little SJ, Holte S, Routy JP, Daar ES, et al. Antiretroviral-drug resistance among patients recently infected with HIV. N Engl J Med. 2002; 347(6): 385-94.

12 Wensing AMJ, van de Vijver DAMC, Asjo B, et al. Analysis from more than 1600 newly diagnosed patients with HIV from 17 European countries shows that 10% of the patients carry primary drug-resistance: the CATCH study. Program and abstracts of the 2nd IAS Conference on HIV Pathogenesis and Treatment; July 13-16, 2003; Paris, France.

01000004273272\1.0\Approved\24-Aug-2004 11:39

13 Chan KCW et al. Prolonged retention of drug resistance mutations and rapid disease progression in the absence of therapy after primary HIV infection. AIDS 17 (8): 1256-1258, 2003.

14 Hirsch MS, Brun-Vezinet F, Clotet B, Conway B, et al. Antiretroviral drug testing in adults infected with HIV-1: Recommendations of an International AIDS Society-USA panel. Clin Infect Dis 2003; 37:113-28.

15 Fauci AS, Bartlett JG, et al. Department of Health and Human Services guidelines for the use of antiretroviral agents in HIV-1 infected adults and adolescents. Nov 10, 2003.

16 Bartlett JA, Johnson J, Herrera G, et al. Abacavir/lamivudine (ABC/3TC) in combination with efavirenz (NNRTI), amprenavir/ritonavir (PI) or stavudine (NRTI): ESS4001 (CLASS) preliminary 48 week results. 14th International AIDS Conference. July 2002, Barcelona, Spain.

17 Montaner JSG, Saag MS, Barylski C, et al. FOCUS Study: Saquinavir qd regimen versus efavirenz qd regimen 48 week analysis in HIV infected patients. 42nd Interscience Conference on Antimicrobial Agents and Chemotherapy; September 2002, San Diego, CA.

18 Robbins G, Shafer R, Smeaton L, et al. Antiretroviral strategies in naïve HIV + patients: comparison of sequential 3-drug regimens (ACTG 384). 14th International AIDS Conference. July 2002, Barcelona, Spain.

19 Staszewski S, Morales-Ramirez J, Tashima K T, Rachlis A, et al. Efavirenz plus zidovudine and lamivudine, efavirenz plus indinavir, and indinavir plus zidovudine and lamivudine in the treatment of HIV-1 infection in adults. N Engl J Med 1999; 341(25):1865-73.

20 Bartlett JG and Gallant JEG. 2003 Medical management of HIV Infection. Johns Hopkins University, Division of Infectious Diseases and AIDS Service; 2003:208-214.

21 Swaden L et al. Efavirenz: what happens in the long term? Ninth Annual Conference of the British HIV Association, Manchester, abstract 09, 2003.

22 Little SJ, Koelsch KK, Ignacio CC, Wong JK, Lie Y, Frost SDW, Richman DD. Persistence of transmitted drug resistant virus among subjects with primary HIV infection deferring antiretroviral therapy. 11th Conference on Retroviruses and Opportunistic Infections, Feb 2004, San Francisco, CA: Abstract 36LB.

0100000427327\1.0\Approved\24-Aug-2004 11:39

23 Palmer S, Boltz V, Maldarelli F, Halvas E, Mican J, Mellors J, Coffin J. Emergence and long-term persistence of NNRTI-resistant variants in patients starting and stopping NNRTI-containing regimens. 11th Conference on Retroviruses and Opportunistic Infections, Feb 2004, San Francisco, CA: Abstract 37.

010000042732721\1.0\Approved\24-Aug-2004 11:39

APPENDICES

APPENDIX A AIDS-DEFINING OPPORTUNISTIC ILLNESSES (OIs)

Clinical Category C Events for Adolescents and Adults (CDC HIV Classification System)

I. PARASITIC INFECTIONS

A *PNEUMOCYSTIS CARINII (PCP)*
A101 Pulmonary PCP, histologically proven
A102 PCP, histologically proven at site other than lungs

B. *TOXOPLASMOSIS*
B101 Toxoplasmosis, clinical diagnosis (of brain only)
B102 Toxoplasmosis, proven by microscopy (of brain or other internal organ other than, liver, spleen, or lymph nodes)

C. *ISOSPORIASIS*
C101 Isosporiasis causing chronic diarrhea >1 month, proven by microscopy

D *CRYPTOSPORIDIOSIS*
D101 Cryptosporidiosis causing chronic diarrhea >1 month, proven by microscopy

II. FUNGAL INFECTIONS

A *CANDIDIASIS*
A201 Candidiasis (esophageal), definitive diagnosis
A202 Candidiasis (esophageal), presumptive diagnosis
A203 Bronquial/pulmonary (lung, trachea), definitive diagnosis

B. *CRYPTOCOCCOSIS*
B201 Cryptococcosis, extrapulmonary, proven by microscopy

FUNGAL INFECTIONS (CONTINUED)

C. *HISTOPLASMOSIS*
C201 Histoplasmosis, extrapulmonary, disseminated, proven by microscopy

D *COCCIDIOIDOMYCOSIS*
D201 Coccidioidomycosis, disseminated, at a site other than, or in addition to, lungs or cervical or hilar lymph nodes, proven by microscopy

III. BACTERIAL INFECTIONS

A *MYCOBACTERIUM*
A301 Mycobacterium tuberculosis, pulmonary
A302 Mycobacterium tuberculosis, extrapulmonary site regardless of pulmonary involvement
A303 Mycobacterium tuberculosis, disseminated, definitive diagnosis, proven by culture

B *NON TB MYCOBACTERIUM INFECTIONS*
B301 Mycobacterium Avium Intracellulare (MAI), extrapulmonary
B302 MAI in blood, proven by culture
B303 MAI colitis, proven by histology and culture
B304 MAI, disseminated, sites other than lungs or cervical or hilar lymph nodes, proven by culture
B305 M. kansasii, extrapulmonary only, proven by culture
B306 M. kansasii, in blood, proven by culture
B307 M. kansasii, colitis, proven by histology and culture
B308 M. kansasii, disseminated at site other than, or in addition to, lungs, or cervical, or hilar lymph nodes, proven by culture

BACTERIAL INFECTIONS (CONTINUED)

C *SALMONELLA*
C301 Salmonella (nontyphoid) bacteremia proven by culture, recurrent

IV. VIRAL INFECTIONS

A *CYTOMEGALOVIRUS (CMV)*
A401 CMV Retinitis
A402 CMV, other than liver, spleen, nodes

B *HERPES SIMPLEX VIRUS (HSV)*
B401 HSV Bronchitis
B402 HSV Pneumonitis
B403 HSV, mucocutaneous (lesions persisting for ≥1 month) proven by microscopy
B404 HSV, mucocutaneous, including HSV esophagitis
B405 HSV encephalitis

C *PROGRESSIVE MULTIFOCAL LEUKOENCEPHALOPATHY*
C401 Progressive Multifocal Leukoencephalopathy

V. NEOPLASTIC DISEASES

A *KAPOSI'S SARCOMA*
A501 Kaposi's sarcoma, mucocutaneous, proven by
A502 Kaposi's sarcoma, mucocutaneous, presumptive
A503 Kaposi's sarcoma, visceral

010000042736651.0\Approved\24-Aug-2004 13:30

AIDS-Defining Opportunistic Illnesses (OIs) (continued)

Clinical Category C Events for Adolescents and Adults (CDC HIV Classification System)

NEOPLASTIC DISEASES (CONTINUED)

B *LYMPHOMA*
B501 Primary brain lymphoma proven by microscopy, Burkitt's, immunoblastic, sarcoma.
B502 Non-Hodgkin B-cell or unknown immunologic phenotype and histology showing small, noncleaved lymphoma
B503 Hodgkin

C *CERVICAL CARCINOMA*
C501 Histologically proven invasive carcinoma of the cervix

VI. **OTHER CONDITIONS**

A *HIV DEMENTIA/MOTOR DEFECTS*
A601 Loss of developmental milestones (pediatric)
A602 Progressive symmetrical motor defects (pediatric)
A603 Adult: Disabling cognitive and/or motor dysfunction interfering with activities.

B *HIV WASTING SYNDROME*
B601 HIV Wasting Syndrome: loss >10% of baseline + chronic diarrhea (>2 loose stools per day during >30 days) or chronic weakness and documented enigmatic fever >30 days.

HIV WASTING SYNDROME (CONTINUED)
B602 715
716

C 717
C601 Progressive symmetrical motor defects (pediatric)
Adult: Disabling cognitive and/or motor

VII. PEDIATRICS

701 Persistent hepatomegaly
702 Persistent splenomegaly
703 Regression of development milestones (consistent regression over at least 3 months)
704 Recurrent or persistent upper respiratory infection, sinusitis or otitis media.
705 Bacterial meningitis, pneumonia, or sepsis (single episode)
706 Candidiasis, oropharyngis for >2 months
707 Cardiomyopathy
708 CMV infection
709 Diarrhea, recurrent or chronic
710 Hepatitis
711 Herpes zoster involving at least 2 distinct episodes or more than one dermatome.
712 Leiomyosarcoma
713 Lymphoid interstiticial pneumonia (LIP)

PEDIATRICS (CONTINUED)

714 Nocardiosis
715 Fever lasting >1 month
716 Varicella, disseminated (ie, complicated chickenpox)
717 Toxoplasmosis of the brain with onset >1 month of age

0100000427366 5\1.0\Approved\24-Aug-2004 13:30

APPENDIX B. LIVER ENZYME WORK-UP

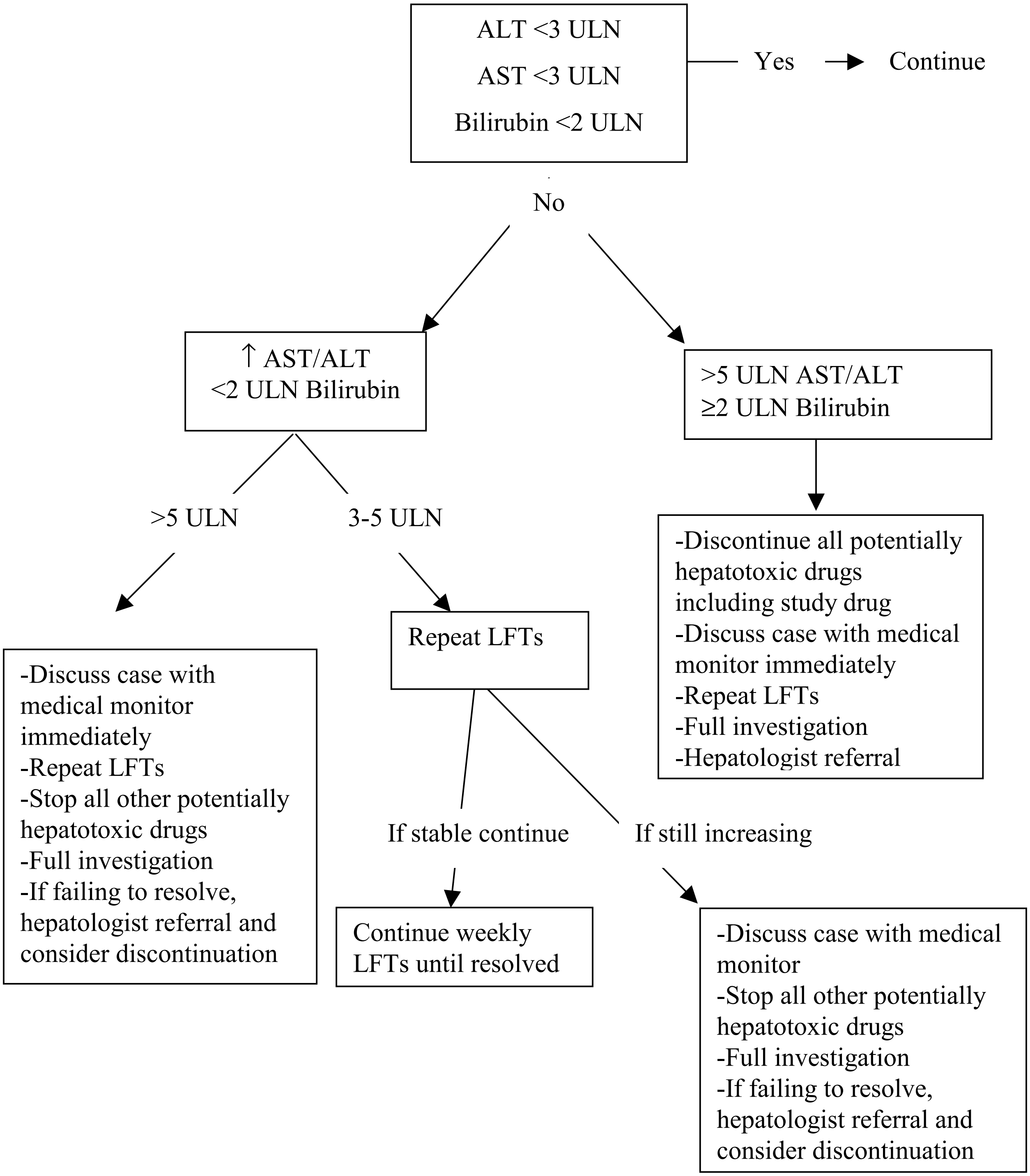

0100000427366 5\1.0\Approved\24-Aug-2004 13:30

APPENDIX C. ANTHROPOMETRIC MEASUREMENT PROTOCOL

Measurements of waist and hip circumference are to be obtained at baseline and Weeks 24, 48, 96, or end of study. Waist and hip circumference measurements are to be done twice and all values recorded to the nearest millimeter in the case report form.

Waist Measurement

The subject should stand with their feet 25 to 30 cm apart with an even distribution of weight. Using the cloth tape measure provided, the measurement is taken midway between the inferior margin of the last rib and the crest of the ileum in a horizontal plane. The subject should take a normal breath, exhale, and the measurement should be taken. The tape should fit snugly but not compress soft tissues. Circumference is measured to the nearest 1 mm.

Hip Measurement

The patient should stand erect with arms at sides and feet together. The hip circumference should be measured at the widest part over the greater trochanters and in a horizontal plane. Circumference is measured to the nearest 1 mm.

Weight

Weight should be measured in light clothes and without shoes. Measurements should be recorded to the nearest 250 grams.

Height

Height should be measured barefoot with head in a horizontal plane and to the nearest 0.1 cm.

01000004273665\1.0\Approved\24-Aug-2004 13:30

APPENDIX D. COLLECTION OF CEREBROSPINAL FLUID

Cerebrospinal fluid (CSF) samples for measurement of UK-427,857 levels will be collected in patients who undergo diagnostic or therapeutic lumbar- or ventricular punctures during the study.

During the lumbar- or ventricular punctures CSF should be collected into two tubes. The first tube will be sent to the local laboratory for measurement of CSF protein, glucose concentrations and cell counts. The second tube will be centrifuged at 1500 g for 10 minutes and supernatant will be stored at –20° C until analyzed for UK-427,857 concentrations by the central laboratory.

On the same day in addition to CSF samples two blood samples will be collected at least 2 hours apart from each other. The detailed description of blood collection is given in section 6.3.

The following data will be recorded in the CRF: method of the CSF collection, time of the last meal, time of the last two UK-427,857 doses, time of the blood and CSF sampling, concentrations of CSF protein, total WBC with differential and RBC.

Because of practical issues associated with lumbar puncture, it is likely that the majority of subjects will have just a single CSF sample collected during the study. Therefore population pharmacokinetic analysis will be used and the plasma concentrations of UK-427,857 will be calculated using the final model for the time of CSF sampling. The penetration of UK-427,857 into CSF will be described by the ratio of CSF and plasma concentrations. All visible bloody CSF samples will be excluded from the final analysis.

0100000427366 5\1.0\Approved\24-Aug-2004 13:30

0100000427366 5\1.0\Approved\24-Aug-2004 13:30

APPENDIX E. ACTG GRADING SEVERITY OF ADULT ADVERSE EVENTS

ABBREVIATIONS: Abbreviations utilized in the Table:

ULN = Upper Limit of Normal	LLN = Lower Limit of Normal
Rx = Therapy	Req = Required
Mod = Moderate	IV = Intravenous
ADL = Activities of Daily Living	Dec = Decreased

ESTIMATING SEVERITY GRADE

For abnormalities NOT found elsewhere on the Toxicity Table, use the scale below to estimate grade of severity:

**GRADE 1**	**Mild**	Transient or mild discomfort; no limitation in activity; no medical intervention/therapy required
**GRADE 2**	**Moderate**	Mild to moderate limitation in activity - some assistance may be needed; no or minimal medical intervention/therapy required
**GRADE 3**	**Severe**	Marked limitation in activity, some assistance usually required; medical intervention/therapy required, hospitalizations possible
**GRADE 4**	**Life threatening**	Extreme limitation in activity, significant assistance required; significant medical intervention/therapy required, hospitalization or hospice care probable

SERIOUS OR LIFE-THREATENING AEs

ANY clinical event deemed by the clinician to be serious or life-threatening should be considered a Grade 4 adverse experience. Clinical events considered to be serious or life-threatening include, but are not limited to:

Seizures, coma, tetany, diabetic ketoacidosis, disseminated intravascular coagulation, diffuse petechiae, paralysis, acute psychosis

MISCELLANEOUS

- When two values are used to define the criteria for each parameter, the lowest values will appear first.
- Parameters are generally grouped by body system.
- Some protocols may have additional protocol specific grading criteria.

ACTG GRADING SEVERITY OF ADULT ADVERSE EVENTS

**PARAMETER**	**GRADE 1**<br>MILD	**GRADE 2**<br>MODERATE	**GRADE 3**<br>SEVERE	**GRADE 4**<br>POTENTIALLY LIFE THREATENING
**HEMATOLOGY**				
Hemoglobin	8.0 g/dL - 9.4 g/dL	7.0 g/dL - 7.9 g/dL	6.5 g/dL - 6.9 g/dL	<6.5 g/dL
Absolute Neutrophil Count	1000 - 1500/mm^3	750 - 999/mm^3	500 - 749/mm^3	<500/mm^3
Platelets	75,000 - 99,000/mm^3	50,000 - 74,999/mm^3	20,000 - 49,999/mm^3	<20,000/mm^3
Prothrombin Time (PT)	>1.0 - 1.25 X ULN	>1.25 - 1.5 X ULN	>1.5 - 3.0 X ULN	>3 X ULN
PTT	>1.0 - 1.66 x ULN	>1.66 - 2.33 x ULN	>2.33 - 3.0 x ULN	>3.0 x ULN
Methemoglobin	5.0 - 10.0%	10.1 - 15.0%	15.1 - 20.0%	>20%
**CHEMISTRIES**				
SODIUM				
Hyponatremia	130 - 135 meq/L	123 - 129 meq/L	116 - 122 meq/L	<116 meq/L
Hypernatremia	146 - 150 meq/L	151 - 157 meq/L	158 - 165 meq/L	>165 meq/L
POTASSIUM				
Hypokalemia	3.0 - 3.4 meq/L	2.5 - 2.9 meq/L	2.0 - 2.4 meq/L	<2.0 meq/L
Hyperkalemia	5.6 - 6.0 meq/L	6.1 - 6.5 meq/L	6.6 - 7.0 meq/L	>7.0 meq/L
PHOSPHATE				
Hypophosphatemia	2.0 - 2.4 mg/dL	1.5 - 1.9 mg/dL	1.0 - 1.4 mg/dL	<1.0 mg/dL
CALCIUM - (corrected for albumin)				
Hypocalcemia	7.8 - 8.4 mg/dL	7.0 - 7.7 mg/dL	6.1 - 6.9 mg/dL	<6.1 mg/dL
Hypercalcemia	10.6 - 11.5 mg/dL	11.6 - 12.5 mg/dL	12.6 - 13.5 mg/dL	>13.5 mg/dL
MAGNESIUM				
Hypomagnesemia	1.2 - 1.4 meq/L	0.9 - 1.1 meq/L	0.6 - 0.8 meq/L	<0.6 meq/L
BILIRUBIN				
Hyperbilirubinemia	>1.0 - 1.5 x ULN	>1.5 - 2.5 x ULN	>2.5 - 5 x ULN	>5 x ULN
GLUCOSE				
Hypoglycemia	55 - 64 mg/dL	40 - 54 mg/dL	30 - 39 mg/dL	<30 mg/
Hyperglycemia (nonfasting and no prior diabetes)	116 - 160 mg/dL	161 - 250 mg/dL	251 - 500 mg/dL	>500 mg/dL
Triglycerides	__________	400 - 750 mg/dL	751 - 1200 mg/dL	>1200 mg/dL

ACTG GRADING SEVERITY OF ADULT ADVERSE EVENTS

**PARAMETER**	**GRADE 1**<br>MILD	**GRADE 2**<br>MODERATE	**GRADE 3**<br>SEVERE	**GRADE 4**<br>POTENTIALLY LIFE THREATENING
Creatinine	>1.0 - 1.5 x ULN	>1.5 - 3.0 x ULN	>3.0 - 6.0 x ULN	>6.0 x ULN
URIC ACID				
Hyperuricemia	7.5 - 10.0 mg/dL	10.1 - 12.0 mg/dL	12.1 - 15.0 mg/dL	>15.0 mg/dL
**LIVER TRANSAMINASE (LFTs)**				
AST (SGOT)	1.25 - 2.5 x ULN	>2.5 - 5.0 x ULN	>5.0 - 10.0 x ULN	>10.0 x ULN
ALT (SGPT)	1.25 - 2.5 x ULN	>2.5 - 5.0 x ULN	>5.0 - 10.0 x ULN	>10.0 x ULN
GGT	1.25 - 2.5 x ULN	>2.5 - 5.0 x ULN	>5.0 - 10.0 x ULN	>10.0 x ULN
Alk Phos	1.25 - 2.5 x ULN	>2.5 - 5.0 x ULN	>5.0 - 10.0 x ULN	>10.0 x ULN
**PANCREATIC ENZYMES**				
Amylase	>1.0 - 1.5 x ULN	>1.5 - 2.0 x ULN	>2.0 - 5.0 x ULN	>5.0 x ULN
Pancreatic amylase	>1.0 - 1.5 x ULN	>1.5 - 2.0 x ULN	>2.0 - 5.0 x ULN	>5.0 x ULN
Lipase	>1.0 - 1.5 x ULN	>1.5 - 2.0 x ULN	>2.0 - 5.0 x ULN	>5.0 x ULN
**CARDIOVASCULAR**				
Cardiac Arrhythmia	________	Asymptomatic; transient dysrhythmia, no Rx req	Recurrent/persistent dysrhythmia; symptomatic, Rx req	Unstable Dysrhythmia, hospitalization, Rx req
Hypotension	Transient orthostatic hypotension, no Rx	Symptoms correctable with oral fluid Rx	IV fluid req, no hospitalization req	Hospitalization req
Hypertension	Transient, increase >20 mm/Hg; no Rx	Recurrent; chronic increase >20 mm/Hg, Rx req	Acute Rx req; outpatient hospitalization possible	Hospitalization req
Pericarditis	Minimal effusion	Mild/mod asymptomatic effusion, no Rx	Symptomatic effusion, pain, EKG changes	Tamponade-Pericard iocentesis OR surgery req
Hemorrhage, blood loss	________	Mildly symptomatic, no Rx required	Gross blood loss OR 1-2 units transfused	Massive blood loss OR >2 units transfused
**GASTROINTESTINAL**				
Nausea	Mild OR transient; reasonable intake maintained	Mod discomfort OR intake decreased for <3 days	Severe discomfort OR minimal intake for ≥3 days	Hospitalization req
Vomiting	Mild OR transient; 2-3 episodes per day OR mild vomiting lasting <1 week	Mod OR persistent; 4-5 episodes per day; OR vomiting lasting ≥1 week	Severe vomiting of all food/fluids in 24 hrs or orthostatic hypotension or IV Rx req	Hypotensive shock OR hospitalization req; IV Rx req
Diarrhea	Mild OR transient; 3-4 loose stools per day OR mild diarrhea lasting <1 week	Mod OR persistent; 5-7 loose stools per day OR diarrhea lasting ≥1 week	Bloody diarrhea; OR orthostatic hypotension OR >7 loose stools/day OR IV Rx required	Hypotensive shock OR hospitalization req

ACTG GRADING SEVERITY OF ADULT ADVERSE EVENTS

PARAMETER	GRADE 1<br>MILD	GRADE 2<br>MODERATE	GRADE 3<br>SEVERE	GRADE 4<br>POTENTIALLY LIFE THREATENING
Oral Discomfort/ Dysphagia	Mild discomfort, no difficulty swallowing	Difficulty swallowing but able to eat and drink	Unable to swallow solids	Unable to drink fluids; IV fluids req
Constipation	Mild	Moderate	Severe	Distention with vomiting
**RESPIRATORY**				
Cough (for aerosol studies)	Transient; no Rx	Treatment associated cough; inhaled bronchodilator	Uncontrolled cough; systemic Rx req	________
Bronchospasm Acute	Transient; no Rx;<br>FEV1 70% - <80% (or peak flow)	Rx req; normalizes with bronchodilator;<br>FEV1 50%-<70% (or peak flow)	No normalization with bronchodilator;<br>FEV1 25% - <50% (or peak flow), retractions	Cyanosis;<br>FEV1 <25% (or peak flow) OR intubated
Dyspnea	Dyspnea on exertion	Dyspnea with normal activity	Dyspnea at rest	Dyspnea requiring O_2 therapy
**NEUROLOGIC**				
Neuro-cerebellar	Slight incoordination OR dysdiadochokinesia	Intention tremor OR dysmetria OR slurred speech OR nystagmus	Ataxia requiring assistance to walk or arm incoordination interfering with ADLs	Unable to stand
Neuro-psych/mood	________	________	Severe mood changes requiring medical intervention	Acute psychosis req hospitalization
Paresthesia (burning, tingling, etc)	Mild discomfort; no Rx req	Mod discomfort; non-narcotic analgesia req	Severe discomfort; OR narcotic analgesia req with symptomatic improvement	Incapacitating; OR not responsive to narcotic analgesia
Neuro-motor	Mild weakness in muscle of feet but able to walk and/or mild increase or decrease in reflexes	Mod weakness in feet (unable to walk on heels and/or toes), mild weakness in hands, still able to do most hand tasks and/or loss of previously present reflex or development of hyperreflexia and/or unable to do deep knee bends due to weakness	Marked distal weakness (unable to dorsiflex toes or foot drop), and mod proximal weakness eg, in hands interfering with ADLs and/or requiring assistance to walk and/or unable to rise from chair unassisted	Confined to bed or wheel chair because of muscle weakness
Neuro-sensory	Mild impairment (dec sensation, eg, vibratory, pinprick, hot/cold in great toes) in focal area or symmetrical distribution	Mod impairment (mod dec sensation, eg, vibratory, pinprick, hot/cold to ankles) and/or joint position or mild impairment that is not symmetrical	Severe impairment (dec or loss of sensation to knees or wrists) or loss of sensation of at least mod degree in multiple different body areas (ie, upper and lower extremities)	Sensory loss involves limbs and trunk

ACTG GRADING SEVERITY OF ADULT ADVERSE EVENTS

**PARAMETER**	**GRADE 1** MILD	**GRADE 2** MODERATE	**GRADE 3** SEVERE	**GRADE 4** POTENTIALLY LIFE THREATENING
**URINALYSIS**				
Proteinuria<br>Spot urine	1+	2 - 3+	4+	Nephrotic syndrome
24 hour urine	200 mg-1 g loss/day OR<br><0.3% OR<br><3 g/l	>1 - 2 g loss/day OR<br>0.3 - 1.0% OR<br>3 - 10 g/l	>2 - 3.5 g loss/day OR<br>>1.0% OR<br>>10 g/l	Nephrotic syndrome OR<br>>3.5 g loss/day
Gross Hematuria	Microscopic only	Gross, no clots	Gross plus clots	Obstructive OR transfusion req
**MISCELLANEOUS**				
Fever<br>oral >12 hours	37.7 - 38.5C OR<br>100.0 - 101.5F	38.6 - 39.5C OR<br>101.6 - 102.9F	39.6 - 40.5C OR<br>103 - 105F	>40.5C OR<br>>105F
Headache	Mild; no Rx req	Mod; or non-narcotic analgesia Rx	Severe; OR responds to initial narcotic Rx	Intractable; or req repeated narcotic Rx
Allergic Reaction	Pruritus without rash	Localized urticaria	Generalized urticaria angioedema	Anaphylaxis
Cutaneous/Rash/ Dermatitis	Erythema, pruritus	Diffuse maculopapular rash OR dry desquamation	Vesiculation OR moist desquamation OR ulceration	ANY ONE: mucous membrane involvement, suspected Stevens-Johnson (TEN), erythema multiforme, necrosis req surgery, exfoliative dermatitis
Local Reaction (2° parenteral Rx - not vaccination or phlebitis skin test)	Erythema	Induration <10 mm OR inflammation OR phlebitis	Induration >10 mm OR ulceration	Necrosis of skin
Fatigue	Normal activity reduced <25%	Normal activity reduced 25-50%	Normal activity reduced >50%; cannot work	Unable to care for self
